# Segmental airway adenocarcinoma-simulating phantom for endoscopic near-infrared optical coherence tomography

**DOI:** 10.1117/1.JBO.30.10.105002

**Published:** 2025-10-07

**Authors:** Eric Brace, Alicia Fung, Adrian Tanskanen, Jeanie Malone, Calum E. MacAulay, Pierre M. Lane

**Affiliations:** a British Columbia Cancer Research Institute, Department of Basic and Translational Research, Vancouver, British Columbia, Canada; b Simon Fraser University, School of Engineering Science, Burnaby, British Columbia, Canada; c University of British Columbia, School of Biomedical Engineering, Vancouver, British Columbia, Canada; d University of British Columbia, Department of Pathology and Laboratory Medicine, Vancouver, British Columbia, Canada

**Keywords:** tissue-mimicking phantoms, endoscopic imaging, endobronchial imaging, optical coherence tomography, near-infrared, optical attenuation coefficient

## Abstract

**Significance:**

There is an unmet need for readily accessible imaging targets to verify whether devices can discriminate lesions from healthy tissue and identify sub-surface vasculature in the small airways.

**Aim:**

Our aim is to develop a phantom that mimics human segmental airway adenocarcinoma *in vivo* for 1310 nm endoscopic optical coherence tomography (OCT) and angiography characterization.

**Approach:**

We develop phantoms using a mixture of agar, intralipid, and coconut oil cured in a 3D printed mold with embedded tubing to mimic vasculature. The parenchyma optical attenuation coefficient (OAC) is calibrated using optical transmission measurements from an agar and intralipid dilution series. Depth-resolved OAC histogram distributions, analysis of variance, and image quality are used to assess repeatability and biofidelity of these phantoms.

**Results:**

Transmission measurements show large increases in OAC when intralipid is cured with agar compared with water-intralipid dilutions. Representative phantom OACs show repeatability within 2.7% and match normal *in vivo* tissue measurements within 16%. Embedded lesion phantoms achieve imaging characteristics of *in vivo* adenocarcinoma. Fluid flow within embedded tubing is visualized with Doppler OCT.

**Conclusions:**

The segmental airway phantoms demonstrate *in vivo* human imaging characteristics, including structural and optical markers of pathological progression—providing a platform for imaging system characterization and optimization.

## 1 Introduction

Endobronchial optical coherence tomography (EB-OCT) involves the deployment of an optical imaging catheter through the working channel of a bronchoscope to capture volumetric images of airway structure with microscopic (∼10 to 40  μm) resolution. EB-OCT has shown promise in airway structural imaging and applications in lung cancer, where there is need for early detection and biopsy guidance.^1^^–^^5^

**Fig. 1:**
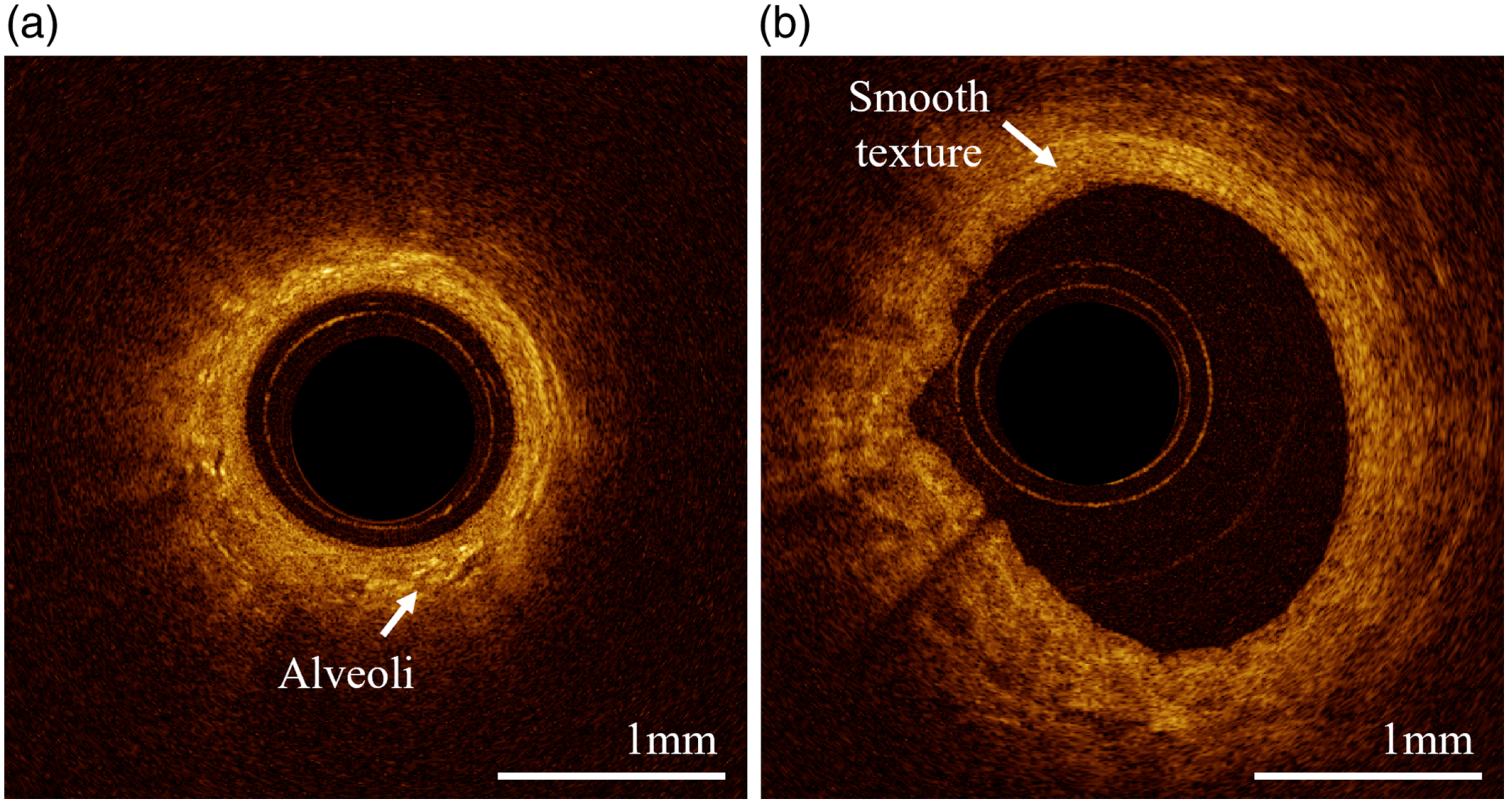
OCT features observed in the airway of a healthy volunteer. (a) Heterogeneous distal portion and (b) smooth texture proximal portion. Both cross-sections are selected from the same pullback.

Implementations of EB-OCT have explored lung tissue optical features through *in vivo* clinical imaging. Healthy segmental airways are characterized by a pseudostratified (one-to-two cell layer thick) hyposcattering columnar epithelial layer, above a highly scattering stroma.^6^^,^^7^ Lumen diameter decreases from the trachea toward the peripheral lung.^8^ A visible change in texture can be observed throughout an OCT pullback in the lower respiratory tract: a heterogeneous appearance due to high presence of alveoli in the distal portion of the pullback [Fig. 1(a)], transitioning to a homogenous texture due to imaging primarily smooth muscle closer to the trachea [Fig. 1(b)]. In the parenchyma, squamous epithelial alveoli (with diameters of ∼200  μm ^9^) generate rounded sharp back reflections when inflated.^10^ When not inflated, these alveoli appear as a small bright object relative to surrounding tissue [Fig. 1(a)].

EB-OCT has been successfully deployed to visualize indicators of carcinoma progression and invasion, such as epithelial thickening, loss of layered architecture, and irregular vasculature.^4^^,^^11^^–^^16^ Clinical functionality of EB-OCT devices, then, need to identify these features and be able to differentiate them from healthy tissue. Phantoms can provide useful benchmarks during development of EB-OCT devices and the algorithms that detect these features; however, there is a lack of lung airway phantoms that model the structural features associated with pathological progression.

OCT leverages low-coherence optical interferometry to measure the difference in optical path length between a sample and a reference. Typical systems utilize a 1310±50  nm swept source laser directed onto target tissue and have resolutions of ∼10  μm axially and ∼40  μm laterally. Backscattering changes that occur in the direction of the beam path (∼2  mm penetration depth into tissue) can then be differentiated and encoded as an axial scan, or A-line. EB-OCT typically uses rotary pullback catheters, consisting of an optical fiber shelled in a torque cable to enable the sample arm to be azimuthally scanned across the inner surface of a lumen to generate a cross-sectional image (B-frame). The catheter can then be retracted, collecting a series of cross-sections to construct an imaging volume. Clinical deployment of EB-OCT necessitates use of single-use sterile catheters, functionally changing the optics along the sample arm for each case. Variations in laser and detector currents, imaging environment temperature, and catheter manufacturing tolerance results in delivered optical power and backscattering intensity response that can vary unpredictably between imaging cases, limiting OCT to qualitative structural/morphological analysis. However, the rate of intensity decay within an A-line is independent of changes in the reference arm; thus, a common method of quantifying OCT data between imaging sessions is to calculate the optical attenuation coefficient (OAC), which governs this exponential rate of decay.^17^^–^^20^ In this work, we use OAC as a quantitative metric to evaluate similarity between OCT of phantoms and tissue.

Optical tissue phantoms normally consist of a transparent matrix, to which scatterers and absorbers are added to mimic the spectral response of tissue.^21^ Efforts have been made to standardize development and deployment of phantoms to ensure comparability to tissue and consistent quality of optical response.^22^^,^^23^ OCT phantoms have historically relied upon silicone, fibrin, or polyvinyl acetate matrices.^24^ The stability of polydimethylsiloxane (PDMS) over time^25^ and tunability in the 500 to 850 nm band^26^ has been explored more recently in retina^27^ and skin^28^ phantoms. However, degassing during curing of these rigid materials can degrade homogeneity, resulting in a high-cost and intensive fabrication approach. Agar-water-gel-based phantoms offer compatibility with biochemical additives in exchange for a limited shelf life, which has been demonstrated to mimic brain, bladder, and lung tissue (532 to 630 nm)^29^ and skin (880 to 1100nm).^30^ Intralipid is a commonly used scattering agent for this approach, due to its predictable scattering response.^31^ Characterization of OAC in agar-intralipid phantoms has been explored in visible wavelengths (550, 650 nm),^32^^,^^33^ but there remains an opportunity for exploration in the near-infrared (NIR) band. NIR OCT phantoms that mimic pathologic progression have been recently developed in colon^34^ and bladder^35^ using Dragon Skin (a polymer commonly used for prop fabrication in film). These layered phantoms utilize changes in mixture ratios to modify optical properties in ways that resemble lesion development. To date, there have been limited attempts to create phantoms mimicking lung morphology in literature. A PDMS murine airway phantom has been developed utilizing 3D printing techniques and imaged using micro-computed tomography;^36^ however there are no examples designed for OCT. Furthermore, work to date has focused primarily on creating phantoms for visible spectrum OCT, with limited development in the NIR range.

Clinical functionality of EB-OCT systems requires the ability to differentiate healthy tissue from that undergoing pathological progression. In this work, we develop an agar-based lung tissue phantom for endoscopic OCT in the NIR range using simple fabrication techniques to produce OAC features similar to that of epithelial thickening. The presented phantom addresses the need for an assessment platform to evaluate EB-OCT device functionality; specifically, we target the capability to differentiate low-scattering lesions from healthy tissue and identify sub-surface vasculature.

## 2 Methods

The phantoms presented here are based on previous work by Ntombela et al.^29^ to develop gel-based phantoms optimized for imaging at 532 and 630 nm. In their work, they describe an agar-gel-based phantom, combined with India ink as an absorptive agent and aluminum oxide ${\mathrm{Al}}_{2}O_{3}$ as a scattering agent. By varying ratios of these ingredients, the authors developed methods to mimic the optical properties of brain, bladder, and lung tissues. Reproducing and collecting OCT of their phantom produced images of a highly absorptive matrix at 1310 nm, with suspended ${\mathrm{Al}}_{2}O_{3}$ aggregates. Using this design as a starting point, we empirically adapted the formulation for 1310 nm OCT. We designed phantoms to mimic airways of the lower respiratory tract (LB8, LB9, RB8, RB9). Our phantoms were designed to replicate the following biological structures and features:

1. Alveoli: regions containing highly scattering objects, with and without enclosure of signal-void areas.
2. Epithelial thickening: lesions appear as regions of lower scattering and attenuation compared with that of surrounding material.
3. Distal-proximal textural variation: transition from heterogeneous to smooth image texture along the pullback direction.
4. Luminal dilation: increasing luminal diameter along the pullback direction.

### 2.1 Attenuation of Intralipid-Water Solutions at 1310 nm

**Fig. 2:**
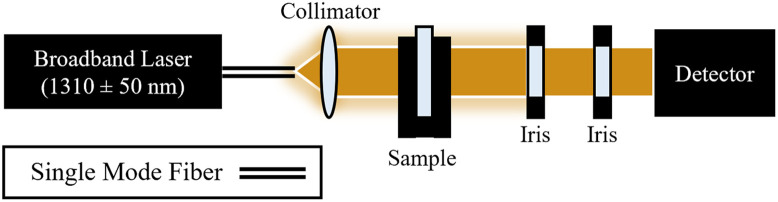
Narrow beam experimental setup.

The OAC of an intralipid-water dilution series was measured to determine the dependence of attenuation on intralipid concentration at 1310 nm. OAC was measured using a narrow-beam method similar to that described in Ref. 37. A 1310±50  nm broadband laser (BBS 1310 B-TS, AFC Technologies, Ramsey, Minnesota, United States) was collimated and directed at a cuvette containing the sample, from which the transmitted beam was shaped by two adjustable irises (SM1D12, Thorlabs Inc., Newton, New Jersey, United States) to reject scattered photons before collection by a detector (S112C, Thorlabs Inc., Newton, New Jersey), as shown in Fig. 2. Irises were adjusted while a water sample was inserted to be at the minimum aperture size without a measured reduction in power.

An intralipid-water dilution series (0 to 0.02 g/mL) was prepared by adding 20% w/v (0.2 g/mL) intralipid (Fresenius Kabi Canada Ltd., Toronto, Canada) to water. The OAC of each dilution was measured using an optical glass cuvette (Hellma® absorption cuvette Z802689 MilliporeSigma Canada Ltd., Oakville, Canada) with pathlength d=1  mm.^38^ Total OAC

$$\mu_{t}=\frac{1}{d}\ln\frac{I_{\text{water}}\;-\;I_{\text{water dark}}}{I_{\text{sample}}\;-\;I_{\text{sample dark}}},$$ (1)

was calculated from four intensities measured at the detector: $I_{\text{sample}}$ is the intensity measured from the sample cuvette, $I_{\text{water}}$ is the intensity measurement from the water cuvette, and the intensities with “dark” subscripts are intensity measurements with the laser blocked. Solutions were loaded into the sample cuvette sequentially from lowest to highest concentration. Between each measurement, the sample cuvette was rinsed 3 times with water, once with isopropyl alcohol, then dried. The water cuvette was not rinsed between readings. All dilutions and measurements (in triplicate) were made on the same day. Intralipid solutions were shaken before being loaded into the sample cuvette to mitigate settling.

### 2.2 Attenuation of Agar-Intralipid-Water Gels

The OAC of several homogeneous agar-intralipid-water gels, prepared with a range of concentrations, were measured to determine (1) the dependence of OAC on intralipid and agar concentration and (2) the OAC impact of intralipid addition before versus after boiling of the agar solution. We measured the OAC of nine gels (intralipid concentrations of 0, 0.008, and 0.016 g/mL, and agar concentrations of 0.01, 0.02, and 0.03 g/mL) using the methodology and apparatus described above. As the cuvettes could not be easily cleaned and reused after curing the gels, we developed a simple multiwell plate to measure gel attenuation.

**Fig. 3:**
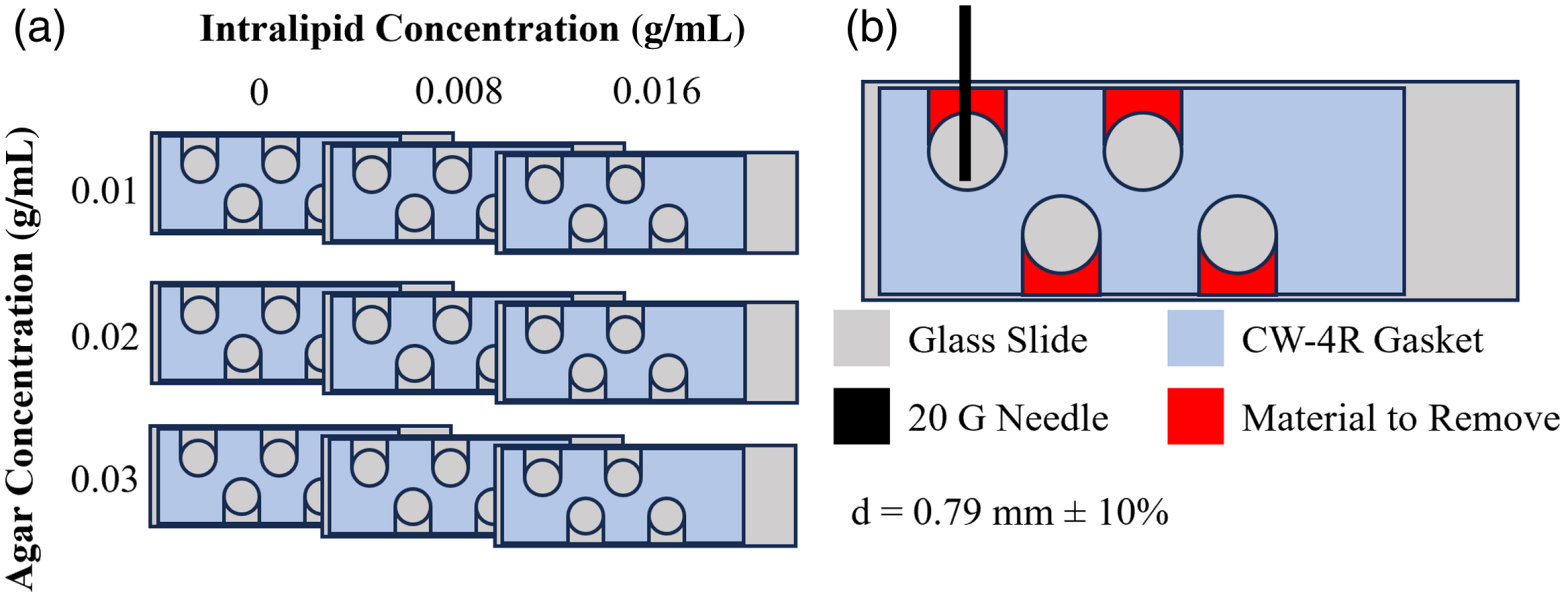
Agar-intralipid-water attenuation experiment. (a) Multiwell plates and intralipid-agar concentration matrix. (b) Multiwell plate construction showing modification to gasket and loading of solution with 20G needle.

The multiwell gel plate (Fig. 3) consisted of a reusable CultureWell Gasket (CW-4R-1.0 Grace Bio-labs, Bend, Oregon, United States) sandwiched between two glass microscope slides. The gasket has four 9 mm diameter holes that define the wells in which the gel solution was loaded and cured, with thickness d=0.79±0.08  mm to provide a sufficiently large change in transmitted optical signal for detection. Material was removed from the gasket using a scalpel to extend wells to the edge of the slide (b). This modification allowed the gel solution to be loaded into the wells from the edge of the assembly using a 20 G blunt needle, thus avoiding the air bubbles captured when a glass slide is simply placed against a nonmodified gasket. The extended well size also increased the clear aperture for the optical transmission measurement. Surface tension was sufficient using this method to allow rotating the slide to fill opposing wells without the solution escaping. The multiwell gel plate enabled four gels to be cured in parallel for repeated measurements, a short pathlength d appropriate to the higher attenuation of the gels, and facilitated cleaning for multiple experiments with the same plate.

Gel solutions were prepared by adding intralipid to an agar-water solution (05039 agar, MilliporeSigma Canada Ltd. Oakville, Canada) in a 100 mL beaker, heating the mixture to 95°C on a hot plate, then dispensing it into wells when cooled to 60°C. The impact of adding intralipid before, or after, boiling agar-water solution is assessed by repeating the experiment under both conditions. The solutions were loaded into the four 9 mm diameter wells of the custom holder. The custom holders containing gel solution were placed immediately in a 4°C fridge to cool for 24 h before transmission data were collected. Measurements were collected over 2 days, with the “before boil” group collected on the first day, followed by the “after boil” group. Between groups, the slides and gaskets were separated to remove the agar-intralipid gels, cleaned in a bath of warm soapy water, allowed to dry overnight, then wiped with optical tissue wetted with isopropyl alcohol.

### 2.3 Gel Formulations

The intralipid-water and agar-intralipid-water attenuation measurements at 1310 nm guided formulation of the gels to provide phantoms with biologically relevant and customizable OACs. The phantoms were prepared from an agar-intralipid-water solution, to which virgin coconut oil (Nutiva, Richmond, California, United States) was added, and then cured in a mold to provide the desired morphology. Two gel formulations (see Table 1 in Sec. 2.3 and Table 1 in the Supplementary Material) were developed to mimic the optical properties (absorption and scattering) and structural appearance of human segmental airway at 1310 nm. The first (0.008 g/mL intralipid) was designed to replicate normal lung tissue. The second (0.002 g/mL intralipid) was designed to emulate the decreased optical attenuation of adenocarcinoma by iteratively reducing the intralipid concentration and comparing to retrospective *in vivo* images. Lower optical attenuation has been observed to be associated with malignant transformation in lung tissue.^39^^,^^40^ A higher concentration of coconut oil can be used to increase signal-void areas within the phantom to simulate regions of emphysematous change. Separation tends to occur, resulting in a layer of concentrated coconut oil at the top of the phantom; however, this can be easily excluded from the calculation regions of interest to minimize the impact on metric evaluation.

**Table 1:** Formulations for normal and lesion lung tissue mimicking phantoms; final agar and intralipid concentrations are shown in brackets.

Reagent	Normal	Lesion
Agar powder	0.5 g (0.01 g/mL)	0.5 g (0.01 g/mL)
Intralipid	2 mL (0.008 g/mL)	0.5 mL (0.002 g/mL)
Coconut oil	3.0 mL	3.0 mL
Deionized water	45 mL	45 mL
Total	50 mL	48.5 mL

### 2.4 Phantom Molds

**Fig. 4:**
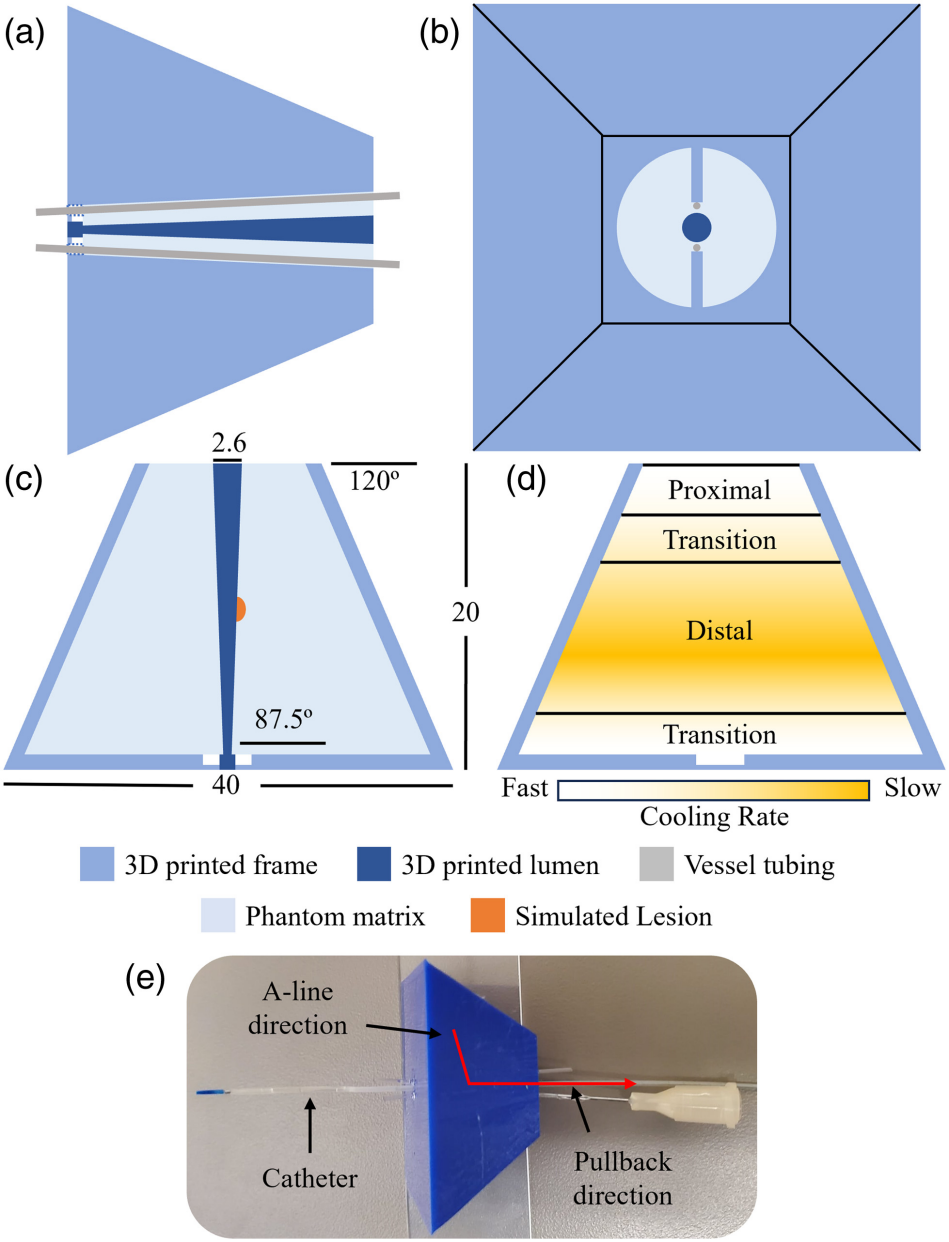
Phantom mold and solution schematic: (a) Tubing placement cross-section, (b) top-down view, (c) dimensioned cross-section showing simulated lesion placement, units in mm, (d) visualization of phantom cooling rate and corresponding biological structures; proximal: smooth texture, agar solidifies with intralipid in solution; distal: heterogeneous texture, intralipid precipitates out of solution before agar solidifies; (e) phantom as-fabricated with catheter inserted.

Molds were 3D printed using fused filament fabrication (P1S 3D Printer, Bambu Lab, Shenzhen, China). A schematic diagram of the mold is shown in Fig. 4 (STL provided in the Supplementary Material). The outer mold had a pyramidal shape to generate differences in cooling rates between the distal and proximal tissue regions of the phantom [Fig. 4(c)]. Variance in thermal mass along the pyramidal structure generates a gradient of setting time, producing alveoli-like structures at the center of the phantom (slow cooling) and smooth muscle-like structures at the surface (fast cooling) [Fig. 4(d)]. During cooling, droplets of intralipid precipitate out of the agar solution, resulting in discontinuities in scattering that appear similar to alveoli. These droplets have diameters beginning from below our system’s resolution and increase along the pullback direction as a function of cooling rate. This mechanism naturally mimics lung tissue inhomogeneity, particularly the index of refraction changes occurring at interfaces between the alveolar wall and the air contained within alveoli. A removable conical structure in the mold simulates increasing airway diameter throughout the pullback [Fig. 4(c)]. Irregularities of the airway lumen are reproduced through the inherent roughness of components produced using a fused filament fabrication 3D printer—larger layer thicknesses could be used to increase this effect. Imaging the phantom is carried out by inserting the catheter through the lumen once the conical structure is removed [Fig. 4(e)], the pullback is initiated from the base of the pyramid mold.

### 2.5 Phantom Construction

**Fig. 5:**
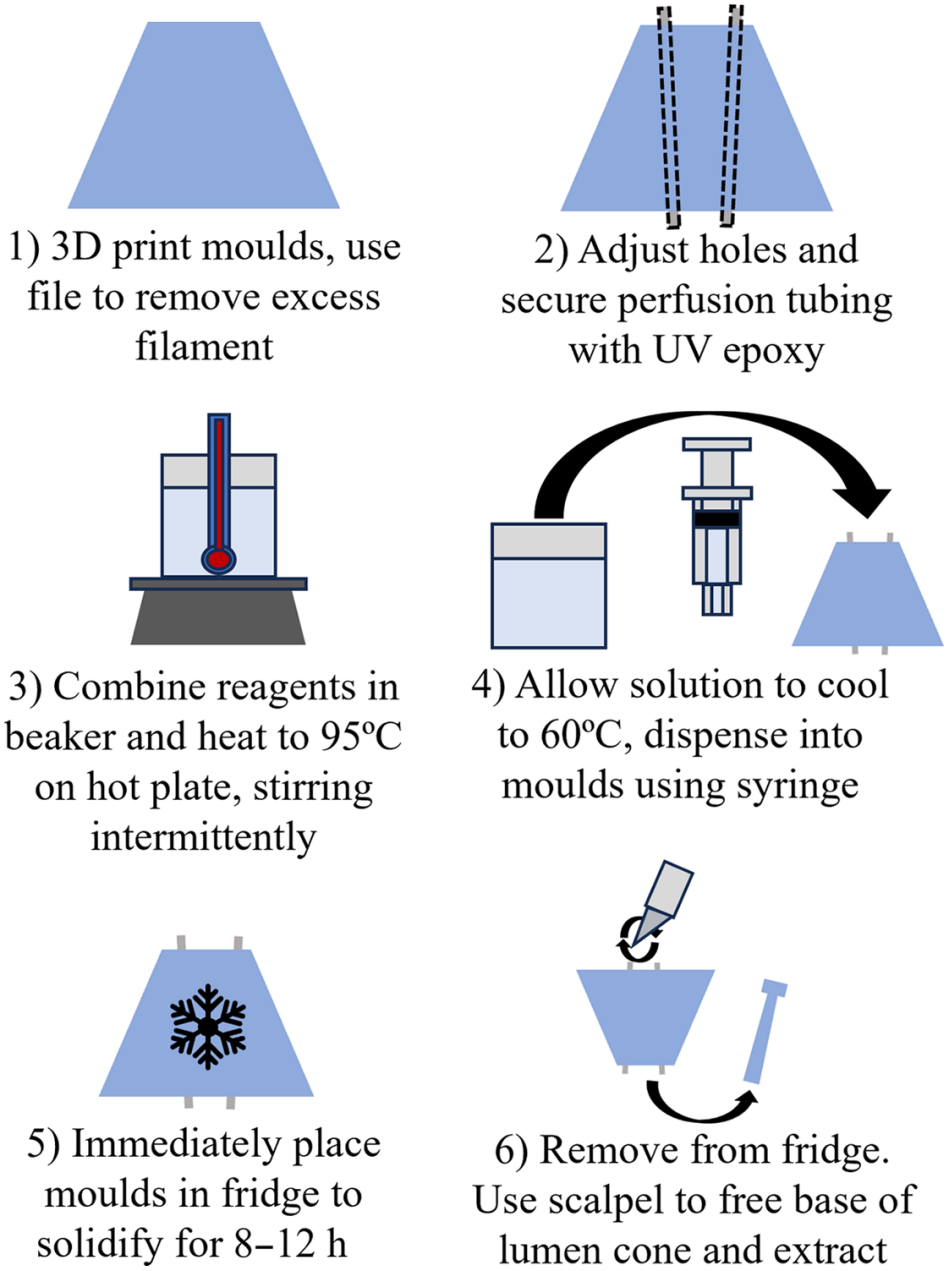
Phantom fabrication process.

The fabrication process involved six steps (Fig. 5, additional details available in the Supplementary Material). Any “stringing” in the molds was removed using a file (Step 1). Holes to accommodate tubing for flow experiments (OCT angiography) were cleaned up using a dental pick. Two 0.53 mm ID (0.08 mm wall) polyethylene terephthalate (PET) tubes (Nordson Medical, Westlake, Ohio, United States) were inserted through the bottom of the mold (Step 2). The tubes were secured with UV-cure epoxy (NOA 63, Norland Products Inc. Jamesburg, New Jersey, United States) at the bottom surface of the mold and to a support rib. One end of the tubes was attached to a blunt needle tip (26 G) for intralipid flow. The reagents were combined in a beaker and heated to 95°C on a hot plate, stirring intermittently to homogenize (Step 3). The mixture was then removed from heat and cooled to 60°C while gently stirring (Step 4). Using a 10 mL syringe with a blunt needle tip (18G), the solution was transferred into the prepared mold and refrigerated (4°C) for at least 8 h to solidify (Step 5). The solution was observed to begin solidifying at 35°C. Once the solution was fully cured, the 3D printed lumen was extracted from the mold using a scalpel (Step 6). The fabrication process described above yields ∼50  mL of solution, sufficient volume to generate four phantoms. An example record for phantom fabrication is provided in Table 2 in the Supplementary Material.

Three types of phantoms were fabricated: “Normal phantoms” were fabricated using 0.008 g/mL intralipid (Table 1, Normal), “all-lesion phantoms” were created using 0.002 g/mL intralipid (Table 1, Lesion), whereas “embedded lesion phantoms” contained a small lesion embedded on the luminal surface of an otherwise normal phantom.

Phantoms with embedded lesions were fabricated by depositing ∼2  mL of lesion solution on the airway luminal mold before filling the mold with normal solution. This was achieved in practice by carefully dispensing a 1 mm diameter droplet directly onto the conical lumen of the internal mold [Fig. 4(c)]. If the solution hardened in the needle, it was submerged in the beaker solution to reheat until able to flow freely. Once the lesion was deposited, the mold was placed in the fridge to cure for 5 min, and the remaining solution was returned to the beaker.

### 2.6 Endoscopic OCT Systems

**Fig. 6:**
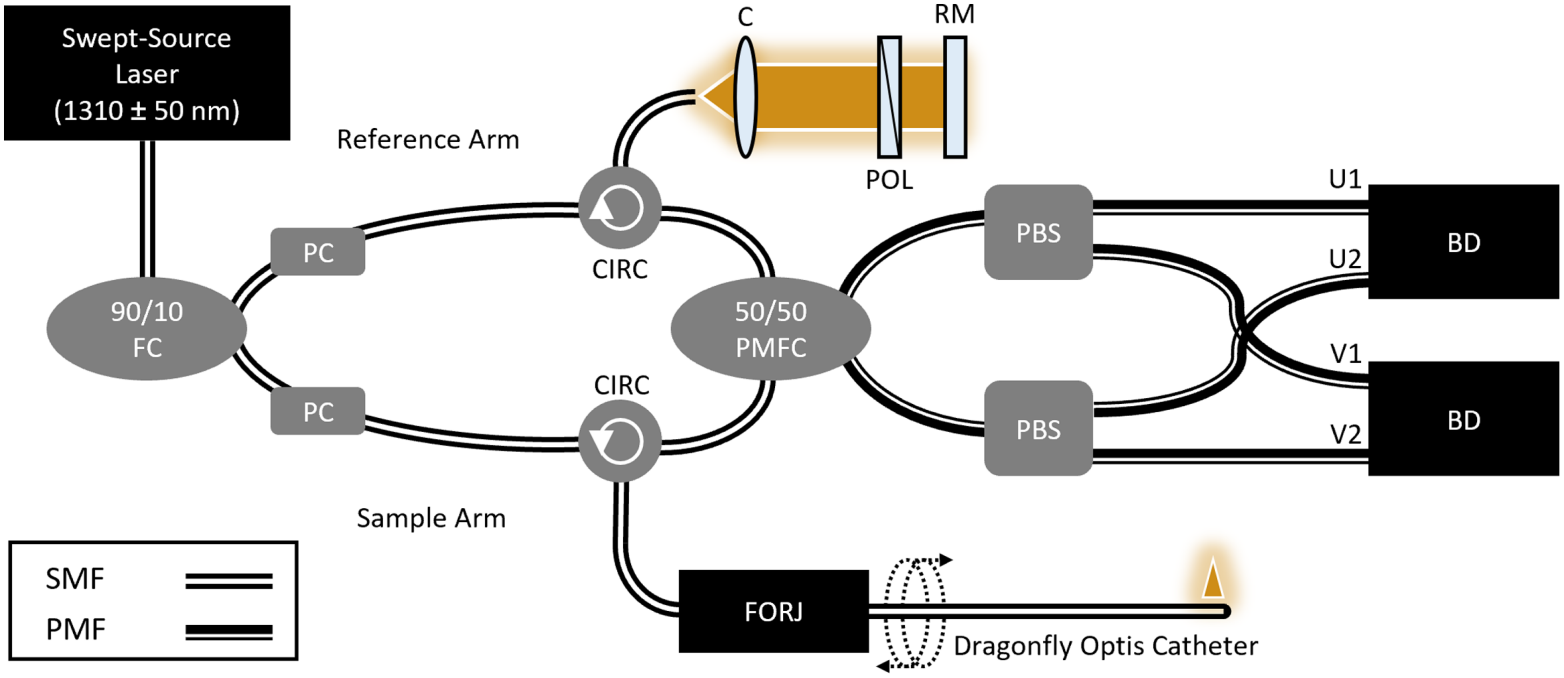
Optical schematic of endoscopic PDD-OCT system. FC, fiber coupler; PC, polarization controller; CIRC, circulator; C, collimator; POL, polarizer; RM, reference mirror; FORJ, fiber optic rotary joint; PMFC, polarization maintaining fiber coupler; PBS, polarization beam splitter; BD, balanced detector; U1, U2, V1, V2 represent orthogonal polarization channels output from the polarization beam splitter; SMF, single mode fiber; PMF, polarization maintaining fiber.

Volumetric images of the phantoms were collected with an endoscopic swept-source OCT system (Fig. 6), which has been described previously.^41^ Images were acquired using commercial 0.9 mm diameter imaging catheters (Dragonfly Optis Imaging Catheter, Abbott, Westford, Massachusetts, United States). The system employed a 1310±50  nm (FWHM) swept-source laser (SSOCT-1310, Axsun Technologies Inc., Billerica, Massachusetts, United States) sweeping at 50.4 kHz. The system employed in this work, used previously in Ref. 41, implemented PPD to mitigate tissue and catheter polarization artifacts; however, polarization diversification is not required to reproduce this work. The spectral interferograms were measured by two 75 MHz balanced detectors (PDB420C, Thorlabs Inc., Newton, New Jersey, United States).

We also present and compare phantom performance against previously collected OCT-AFI of the lung. A second multimodal OCT system that collected autofluorescence images (AFI) in addition to OCT, referred to here as the OCT-AFI system, was employed to collect some of the *in vivo* OCT used for comparison in this study. This system has also been described previously.^42^ Briefly, this system employs the same OCT subsystem as the OCT-only system described above, but without PDD detection. The imaging catheter used with the OCT-AFI was fabricated using double clad fiber to support fluorescence detection, and consequently, the OCT images show some multipath artifacts.

### 2.7 OCT Volume Collection and Processing

OCT phantom volumes were acquired by inserting the imaging catheter into the lumen of the phantom and pulling back the optical core of the catheter from distal to proximal. Pullbacks were acquired at 1 mm/s, and 1792 A-lines were collected per B-scan. Regions of interest were selected manually to exclude regions of 3D printed material or poor catheter-phantom contact, generating ∼350 contiguous B-scans per phantom (∼85% of phantom length, 1.7 cm). Voxel intensity was averaged with one adjacent pixel on either side in the rotational and pullback directions. An example record for phantom imaging is provided in Table 3 in the Supplementary Material.

OAC images were calculated from each B-scan in the volume using the depth-resolved attenuation method described by J. Liu et al.^18^ As part of the calculation, a binary mask was applied to each B-scan, setting all signal intensities below 5 dB from the noise floor to 0. Image processing and analysis were performed with MATLAB ver. R2024b.

Quantitative assessment of OCT data repeatability (batches of similar phantoms) and similarity (phantoms versus *in vivo*) in this work is carried out through comparison of OAC histogram distributions. Histograms of OAC, $h(k)=\frac{1}{N}m_{k}$, were generated for each volume, where $m_{k}$ is the number of counts in bin k∈{1,2,…,K}, and $N=\sum m_{k}$ is the number of voxels in the volume. The histogram bin width was fixed at $0.1\;{\mathrm{mm}}^{-1}$. We compared the distributions of OAC, of two or more groups of volumes, by calculating the mean and range (minimum and maximum) of the histograms in each group. For a group of L volumes, fabricated or imaged under similar conditions, we define a group-wise histogram to have four components,

$$h_{\text{min}}(k)=\frac{1}{N}\underset{l}{\min}\;m_{k,l}$$ (2)

$$h_{\text{mean}}(k)=\frac{1}{\mathrm{NL}}\sum_{1}^{L}m_{k,l}$$ (3)

$$h_{\text{max}}(k)=\frac{1}{N}\underset{l}{\max}\;m_{k,l}$$ (4)

$$k_{\text{mode}}=\underset{k}{\arg\;\max}\sum_{1}^{L}m_{k,l}$$ (5)

where the matrix $m_{k,l}$ represents the number of counts in bin k of volume l∈{1,2,…,L}. Group-wise histograms were used to compare one or more groups of phantom volumes created under different conditions (i.e., different formulations or batches) or to compare a group of phantom volumes with a group of tissue volumes acquired *in vivo*. A group-wise histogram is presented with a central solid line representing the mean and a shaded area of the same color representing the range (minimum and maximum). A vertical line denotes the mode of the histogram, which is used as the metric for comparison in this work as it is not biased by edge effects or filtering. The inter-batch variability in OAC was calculated from the modes of the averaged data by dividing each mode difference by the maximum of each pair of modes.

### 2.8 Repeatability Experiment

**Fig. 7:**
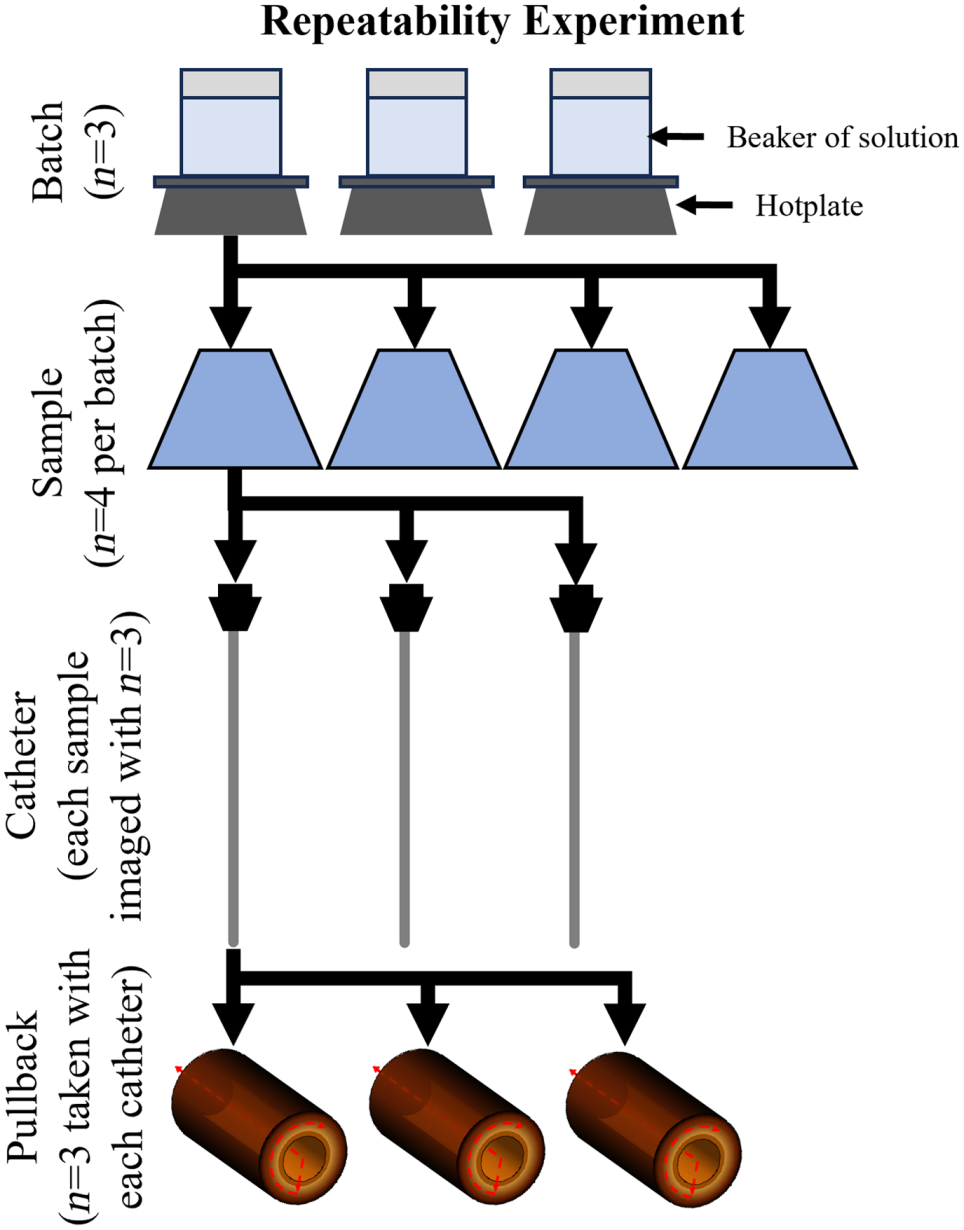
Factorial experiment to assess the repeatability of phantom fabrication and imaging. The experimental design includes four factors (batch, sample, catheter, pullback) and provides 3×4×3×3=108 observations.

We investigated the repeatability of phantom fabrication and imaging using a factorial experiment. The experimental design (Fig. 7) investigated four factors: batch, sample, catheter, and pullback. A batch refers to a group of phantoms that were all prepared from the same mixture of reagents (Table 1, Normal), sequentially, over a period of ∼3  h. A sample refers to a single phantom instance produced in a batch. Samples were imaged using multiple commercial imaging catheters, all functionally identical. Finally, multiple pullbacks (repeated measurements) were acquired using each imaging catheter. In our experiment we prepared three batches of phantom solution, fabricated four samples from each batch, imaged each sample with three different imaging catheters, and three individual pullbacks were collected using each imaging catheter, resulting in the collection of 108 volumes. Each pullback has a collection time of 30 s. The samples and batches were images sequentially with each catheter, with approximately 1 h elapsing before a given sample was re-imaged with the proceeding catheter. Samples were rotated 90 deg around the axis of the imaging catheter between pullbacks to reduce potential bias owing to initial catheter orientation and local discontinuities within the sample.

Histograms of OAC were determined for each volume. Group-wise histograms (min, mean, max, and mode) were calculated for each group using Eqs. (2–5). Repeatability was assessed graphically by plotting the group-wise histograms on the same axis to compare attenuation distribution by factor. Finally, the representative mode OAC was calculated for each of the 108 pullback histograms. The first peak of the OAC histogram (determined to result from edge effect) was removed before calculating its representative mode value by setting all histogram bins with attenuation $\mu_{t}<1\;{\mathrm{mm}}^{-1}$ to 0. A four-way analysis of variance (ANOVA)^43^ was used to investigate the effect of batch, sample, catheter, and pullback (factors) on representative mode OAC (dependent variable). P-values less than 0.05 were considered significant, indicating the factor has a large impact on the mode OAC.

### 2.9 Lesion Phantom Imaging

A group-wise histogram of OAC representing the all-lesion type of phantom was computed and compared with that of the normal phantom type. To calculate the all-lesion histogram, four all-lesion phantoms (Table 1, Lesion) fabricated from the same batch were imaged using a single commercial imaging catheter. Three pullbacks (repeat measurements) were collected from each phantom providing 12 volume observations. The group-wise histogram was calculated from the 12 volumes using Eqs. (2–5). Volumes from four embedded lesion phantoms were collected using the same procedure to provide twelve volumes.

### 2.10 *In Vivo* Airway Imaging

Endobronchial OCT from two previous imaging studies were compared with the phantom OCT. Presumed-normal lung OCT was acquired *in vivo* from a healthy volunteer^44^ using the OCT-PDD system. Volume histograms of OAC were calculated from nine pullbacks, collected from multiple subsegmental airways in the left and right lower lobes (LB8, LB9, RB8, and RB9). A group-wise histogram was calculated from the nine observations using Eqs. (2–5). Two pullbacks (repeated measurements) were collected from a second patient^45^ with suspected lung cancer using the OCT-AFI system and custom double-clad fiber (DCF) imaging catheters. Biopsies from the same airway confirmed adenocarcinoma with lesions also visible on OCT and AFI. Both imaging studies were approved by the University of British Columbia Research Ethics Board (H14-00695 and H17-02439).

### 2.11 Phantom Angiography

**Fig. 8:**
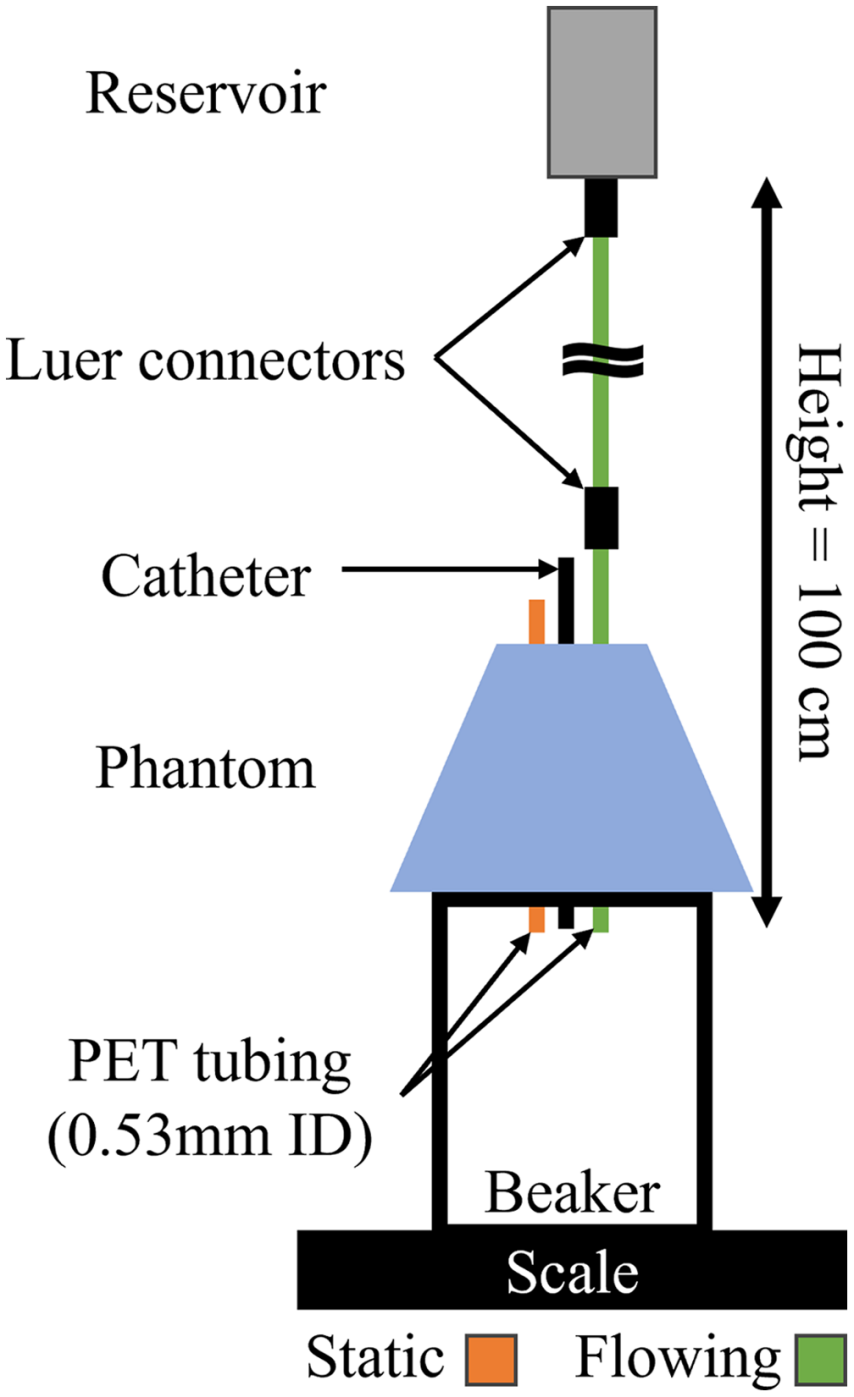
Flow experiment showing flowing and static 0.02 g/mL intralipid dilution through 0.53 mm ID PET tubing embedded in phantom. Height is measured from the level of intralipid solution to the output of the phantom tubing.

Intralipid was flowed through the tubes of an embedded lesion phantom to simulate vessel physiology and demonstrate the collection of OCT angiography from the phantom. A reservoir of dilute intralipid solution (0.02 g/mL) was connected to one of the flow tubes (0.53 mm ID) embedded in the phantom (Fig. 8). The intralipid solution was gravity-fed to provide a flow rate of 0.8 mL/min, a value based on vasculature of the same diameter.^46^ The contralateral tube was filled with static intralipid of the same concentration. Flow rate was validated by placing the phantom on a beaker and measuring the mass flow rate using a scale.

OCT volumes were acquired from the phantom using the PDD-OCT system, with the same acquisition settings (A-lines per B-scan and pullback rate) described above. Images were post processed to visualize flow using Doppler OCT as previously described.^47^ Pixels with phase |ϕ|<0.15π were masked for display.

## 3 Results

### 3.1 Attenuation of Intralipid-Water Solutions and Agar-Intralipid-Water Gels

**Fig. 9:**
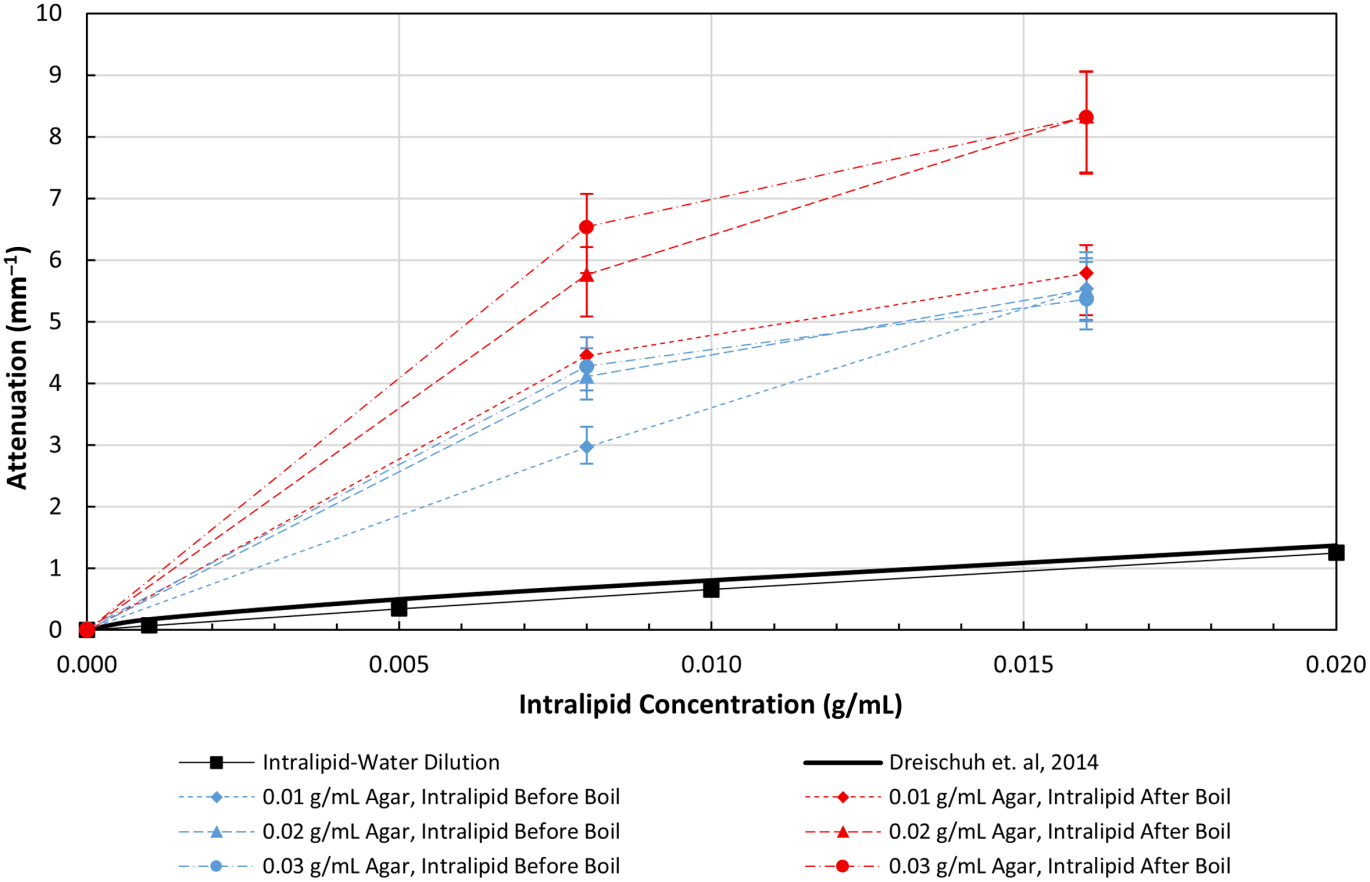
Attenuation coefficient versus intralipid concentration for intralipid-water solutions and agar-intralipid-water gels. Error bars indicate calculated attenuation variability due to the thickness tolerance of the multiwell plates.

Our measurements of the OAC of intralipid-water solutions at 1310 nm, and those reported previously at 1326 nm by Dreischuh et al.,^48^ are plotted versus intralipid concentration in Fig. 9. There is good agreement between the two curves: the slopes are equivalent (62.3 versus $66\;{\mathrm{mm}}^{-1}/g/\mathrm{mL}$); however, Dreischuh’s curve is offset from ours owing to the difference in measurement methodology. We measure attenuation relative to water, whereas Dreischuh’s measurement is absolute (the OAC of water at 1326 nm is $∼0.2{\;\mathrm{mm}}^{-1}$).^49^^,^^50^

Figure 9 also shows OAC for agar-intralipid-water gels versus intralipid concentration, with curves shown for 0.01, 0.02, and 0.03 g/mL agar concentration for intralipid added to the agar solution before (blue curves) or after (violet curves) boiling. Agar-water gels are found to be nonattenuating for concentrations <0.03  g/mL in water. Cured agar-intralipid-water gels show attenuation greater than intralipid-water solutions; this effect is more pronounced at agar concentrations of 0.02 and 0.03 g/mL than at 0.01 g/mL. Furthermore, boiling the solution with intralipid present appears to decrease the attenuation compared with adding intralipid after boiling the agar-water solution. Additional experiments would be required to fully characterize the impact of agar-intralipid interactions on attenuation.

### 3.2 Comparison of Phantom Attenuation by Intralipid Concentration

**Fig. 10:**
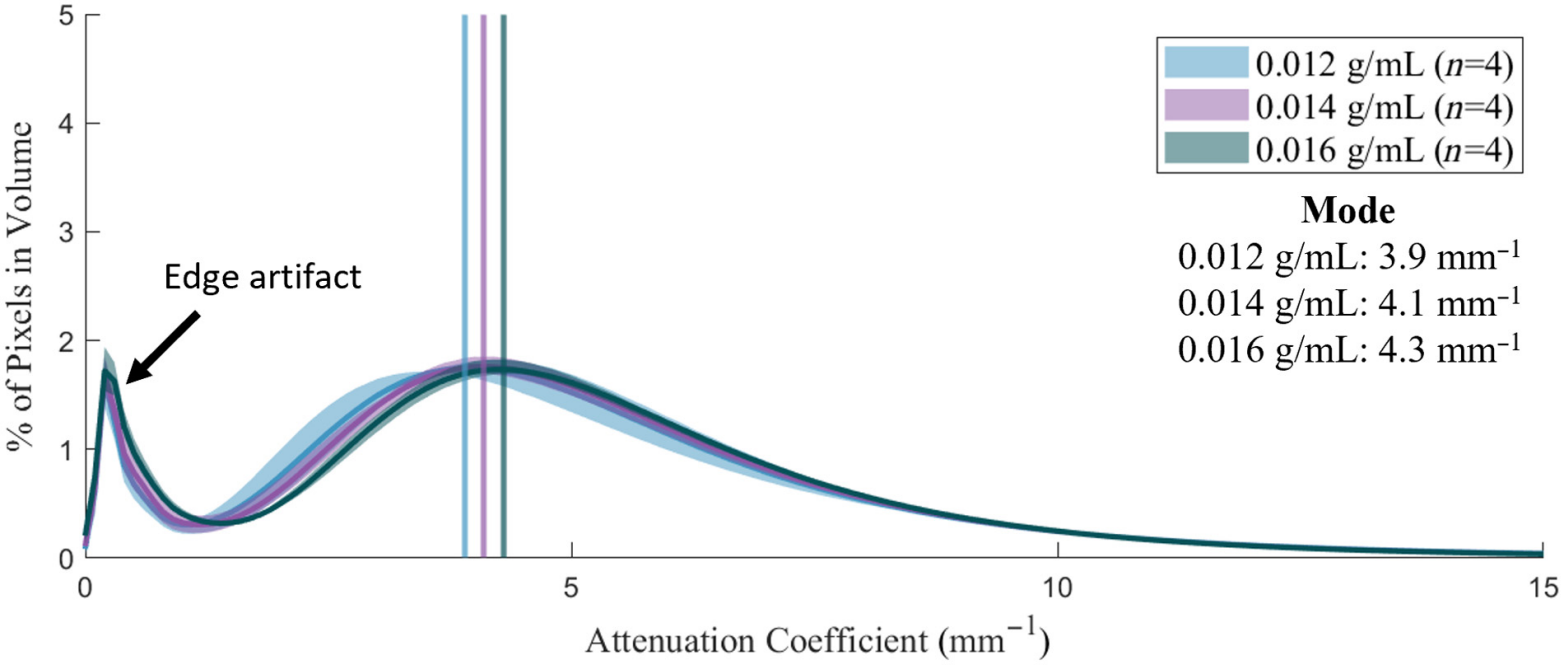
Distribution of attenuation coefficient for phantoms with increasing intralipid concentration. Group-wise histograms have a mean value (solid line) and range (shaded region). Vertical lines indicate distribution modes.

We created three groups of agar-intralipid-water phantoms, with increasing intralipid concentration, and imaged them to validate the expected increase in OAC. The gels were fabricated with 0.012, 0.014, and 0.016 g/mL intralipid and 0.02 g/mL agar. Group-wise histograms were calculated from the three volume groups, and each group contained four phantoms. The distributions of OAC in Fig. 10 have two arithmetic modes: the first mode is an artifact owing to the system point spread function, whereas the second (representative) mode represents the distribution of OAC in the phantom. The representative modes at 3.9, 4.1, and $4.3\;{\mathrm{mm}}^{-1}$ increase with intralipid concentration as expected.

**Fig. 11:**
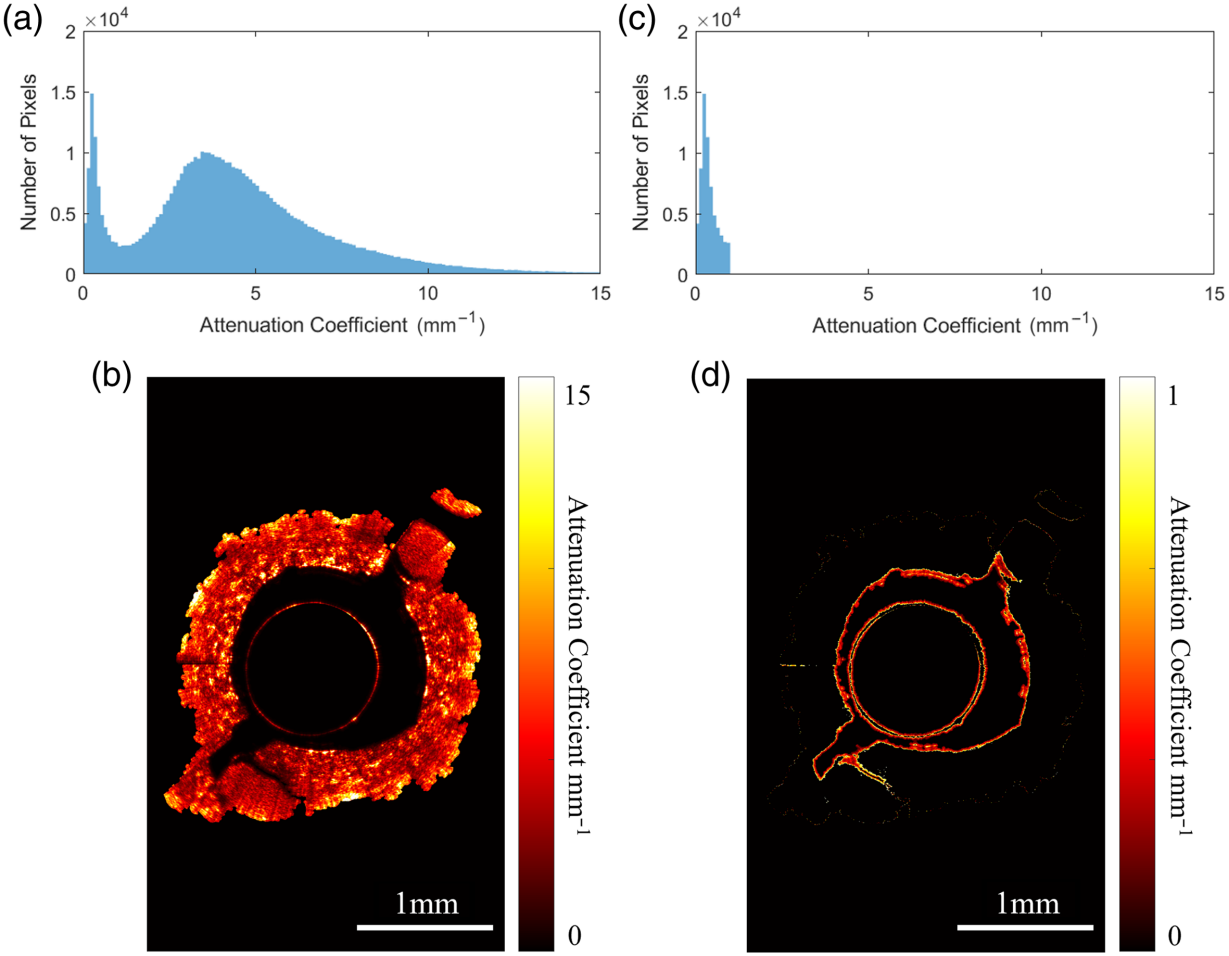
Spatial location of pixels contributing to the first histogram mode (edge artifact): Original bimodal distribution and OAC image (left); distribution and image with threshold ($\mu_{t}<1\;{\mathrm{mm}}^{-1}$) applied (right).

The histograms of OAC derived from both phantom and *in vivo* airways exhibit a bimodal distribution [Figs. 11(a) and 11(b)]. Applying a threshold $\mu_{t}<1\;{\mathrm{mm}}^{-1}$ to select the first mode reveals OACs in this range to be located at the edges of the airway wall (and imaging catheter) [Figs. 11(c) and 11(d)]. We hypothesize this effect is due to the system’s 3D point spread function interacting with the discontinuity of OAC at air–tissue boundaries. Convolution of the point spread function with OAC algorithm output at these boundaries produces a smooth distribution of values that do not represent the true OAC on either side of the boundary. This effect does not appear at the bottom of the image as there is a gradual loss of signal rather than a hard tissue boundary.

**Fig. 12:**
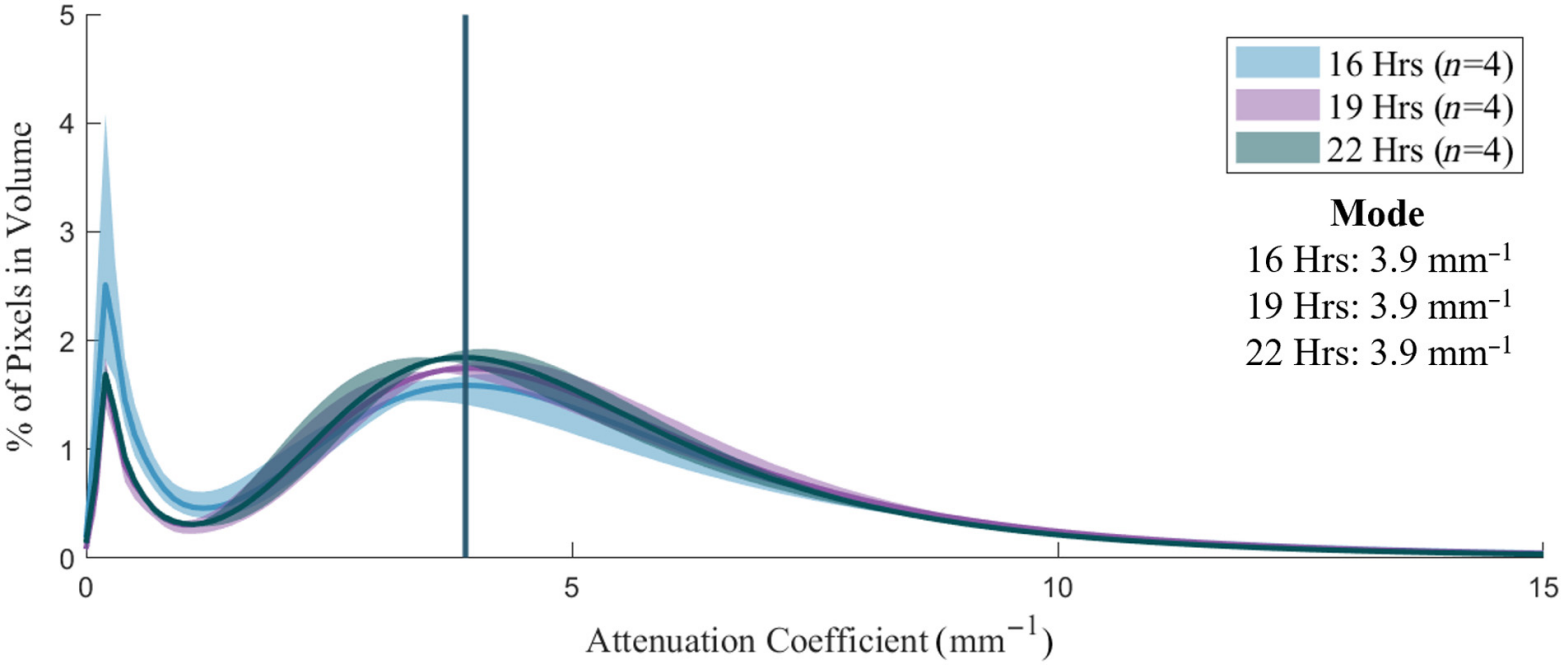
Attenuation coefficient distribution comparing impact of increased time between fabrication of phantoms and imaging. 0.012 g/mL intralipid phantom shown.

Experiments exploring the length of time between phantom fabrication and imaging showed a redistribution of histogram counts from the first mode to the second with longer time intervals (Fig. 12). Exposed surfaces of the phantoms desiccate over time, reducing the amount of liquid present. The result is a decrease in specular reflections at the surfaces and improved coupling of light into the phantom. This may result in changes to the distribution of OAC, with lower water content corresponding to a shift from low to high OAC, when comparing samples with a longer time interval before imaging. Due to this artifact, calculation of mean OAC for an image will underestimate the true value unless these pixels are filtered out. Use of mode as a metric for comparison can avoid this issue, however the artifact may have a higher count than the primary phantom response. Consequently, filtering is required when using either approach.

### 3.3 Repeatability

The repeatability of phantom fabrication and imaging was measured using a factorial experiment with four factors: batch, sample, catheter, and pullback. Results were assessed qualitatively using group-wise OAC histograms and quantitatively using a four-way ANOVA.

**Fig. 13:**
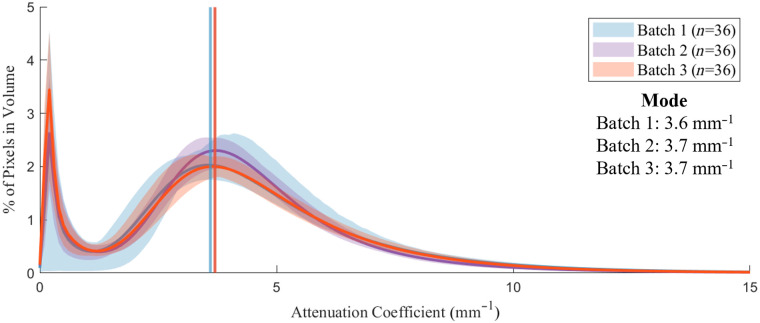
Attenuation distributions from three phantom batches of the multifactorial experiment. Overlapping distributions suggest repeatability of the fabrication process.

Figure 13 shows group-wise histograms for three phantom batches, each with 36 observations. Again, the distributions are bimodal: the first mode is introduced by the edges of the phantom, whereas the second represents the true distribution of OAC. The three batches show overlapping distributions (the vertical line indicating batch 2 is entirely overlapping with the line from batch 3), suggesting repeatability of the fabrication technique. The calculated inter-batch variability was less than 2.7%. There was a 17% difference between the minimum and maximum modes of all pullbacks within the data set.

The results from the four-way ANOVA are shown in Table 2. Catheter, batch, and sample are equally significant factors of variability, whereas pullback is not. The batch:sample interaction term is also significant. Catheter variability is comparable to that of phantom variability, suggesting the optical differences between catheters is similar to variations between phantom batches and samples. Therefore, we conclude that repeatability is within the imaging system’s capability to differentiate between variables in phantom fabrication.

**Table 2:** Phantom fabrication repeatability four-way ANOVA.

Source	Sum of squares	Degrees of freedom	Mean Sq. (SS/DoF)	F (MS/errorMS)	p-value
**Catheter**	**0.514**	**2**	**0.257**	**25.91**	<0.0001
**Batch**	**0.502**	**2**	**0.251**	**25.29**	<0.0001
**Sample**	**0.67**	**3**	**0.223**	**22.52**	<0.0001
Pullback	0.007	2	0.003	0.34	0.716
Catheter:batch	0.071	4	0.018	1.79	0.14
Catheter:sample	0.037	6	0.006	0.63	0.709
Catheter:pullback	0.048	4	0.012	1.20	0.317
**Batch:sample**	**0.954**	**6**	**0.159**	**16.03**	**<0.0001**
Batch:pullback	0.013	4	0.003	0.34	0.853
Sample:pullback	0.027	6	0.004	0.45	0.844
Error	0.674	68	0.009	—	—
Total	3.517	107	—	—	—

Note: Factors with a p-value <0.05 are bolded.

### 3.4 Attenuation Contrast between Normal and All-Lesion Phantoms

**Fig. 14:**
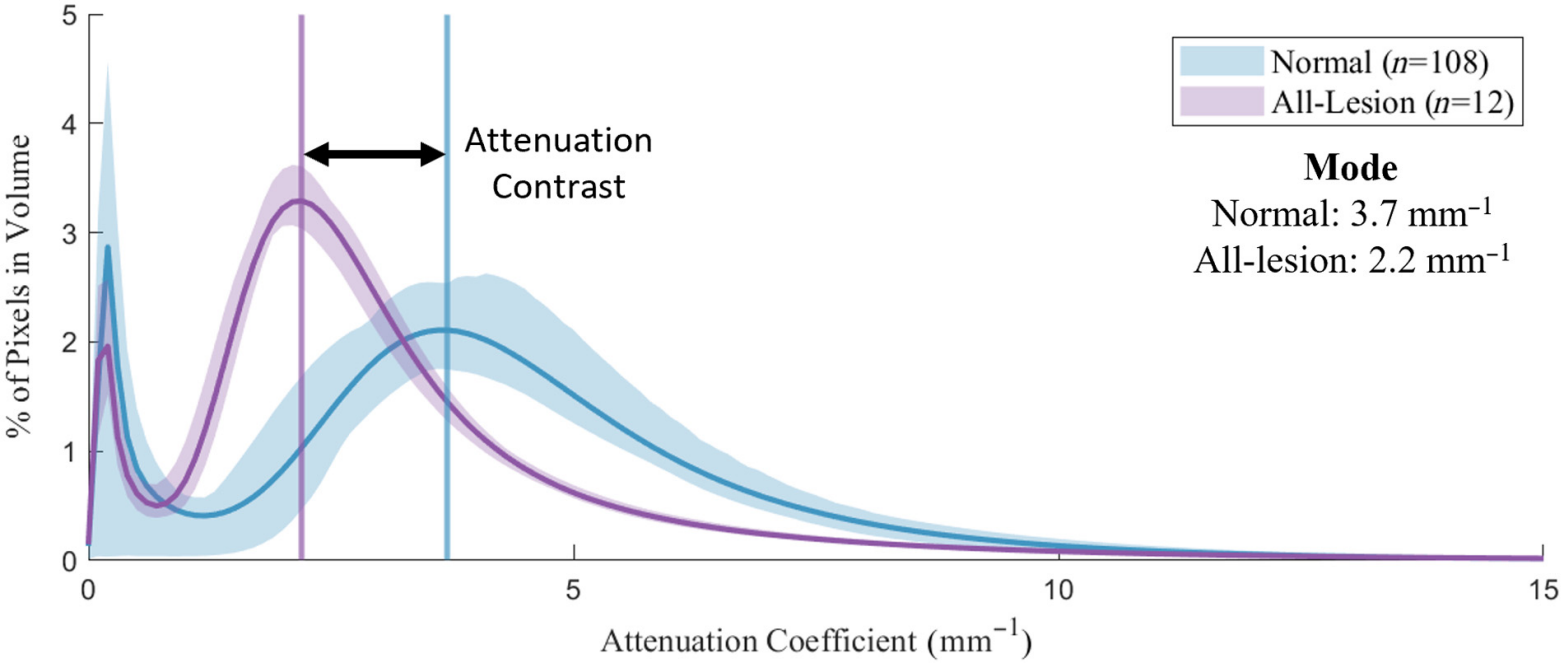
Attenuation distributions for normal and all-lesion phantoms.

OAC histograms for normal (n=108) and all-lesion phantom (n=12) groups were compared to quantify attenuation contrast. As shown in Fig. 14, the normal phantom group (0.008 g/mL intralipid) provides higher OAC (mode $3.7\;{\mathrm{mm}}^{-1}$) compared with the all-lesion phantom group (0.002 g/mL intralipid), which provides lower OAC (mode $2.2\;{\mathrm{mm}}^{-1}$). The attenuation contrast, defined as the difference in OAC mode between the normal and all-lesion phantom groups, is thus $1.5\;{\mathrm{mm}}^{-1}$. Finally, the width of the two modes responsible for phantom attenuation differs between the groups: the width of the normal mode ($3.6\;{\mathrm{mm}}^{-1}$ FWHM) is wider than that of the all-lesion mode ($2.2\;{\mathrm{mm}}^{-1}$ FWHM).

### 3.5 Attenuation Similarity between Normal Phantom and Normal *In Vivo* Airway

**Fig. 15:**
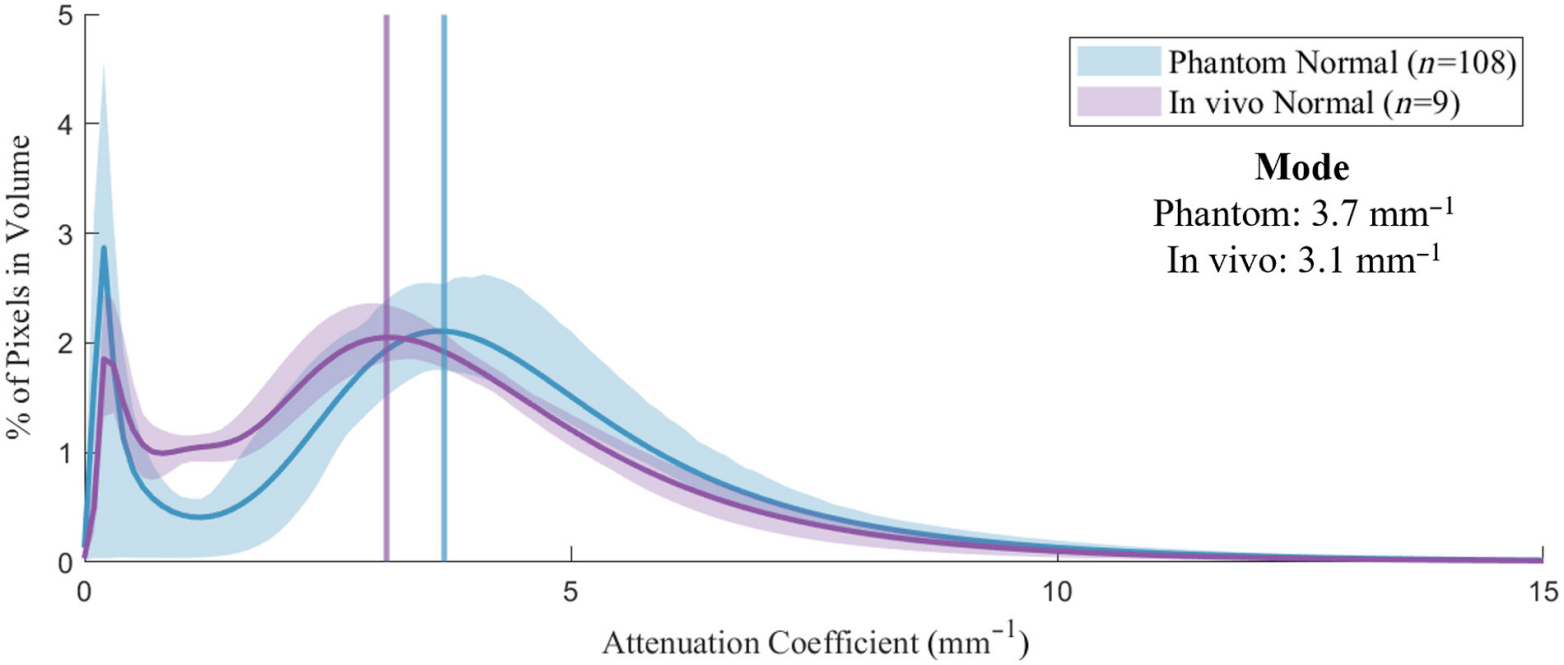
Attenuation distribution for normal phantom and normal *in vivo* airway.

OAC histograms for the normal phantom group (n=108) and a group of normal airway pullbacks acquired *in vivo* (n=9) were compared to confirm the distributions were similar. As illustrated in Fig. 15, there is considerable overlap between the two distributions. The OAC of the normal phantom group (mode $3.7\;{\mathrm{mm}}^{-1}$) was slightly higher than the *in vivo* airway group (mode $3.1\;{\mathrm{mm}}^{-1}$), a relative difference of 16%. The *in vivo* airway group shows increased OAC around $2\;{\mathrm{mm}}^{-1}$, due to intensity variations throughout the tissue layer architecture, which was not simulated in the phantoms. There was a 14% difference between the minimum and maximum modes of all pullbacks within the *in vivo* data set.

### 3.6 Embedded Lesion Contrast

**Fig. 16:**
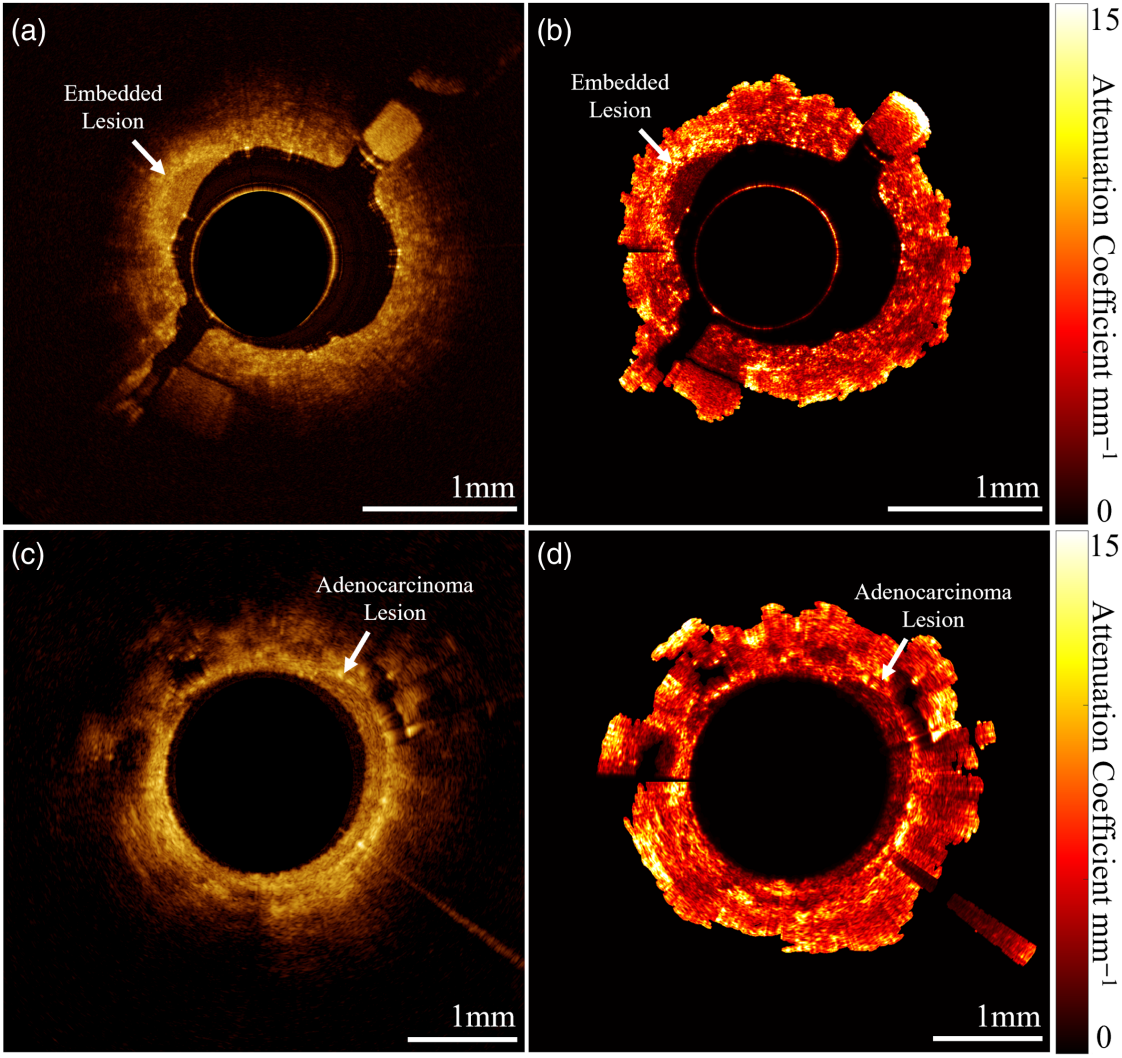
Endobronchial OCT and depth-resolved attenuation image of an embedded lesion phantom (a, b) and patient airway with biopsy-confirmed adenocarcinoma, acquired *in vivo* (c, d) (Video 2, MP4, 3.51 MB [URL: https://doi.org/10.1117/1.JBO.30.10.105002.s2]; Video 4, MP4, 9.59 MB [URL: https://doi.org/10.1117/1.JBO.30.10.105002.s4]).

OCT volumes collected from an embedded lesion phantom were compared with volumes collected *in vivo* from a patient airway (LB9) with a biopsy-confirmed adenocarcinoma. Figure 16 shows B-scans from each volume illustrating the embedded lesion of the phantom [Fig. 16(a)] and the adenocarcinoma from the patient airway [Fig. 16(c)]. Depth-resolved OAC images processed from the B-scans are shown in Figs. 16(b) and 16(d).

The B-scans, both located distally in the airway segment, show granular textural features corresponding to alveoli, which are visible in both phantom and patient airways with comparable optical penetration depth of ∼0.5  mm. In addition, the phantom and *in vivo* lesions both show high OCT contrast with its surrounding tissue. The lesions show a region of lower OAC relative to surrounding tissue, corresponding to epithelial thickening, generating a distinct visible boundary in phantom and patient airways.

We observed regions of high OAC immediately outside the low attenuation lesion, in both the OAC images collected from the embedded lesion phantoms and *in vivo* airway with adenocarcinoma. We suspect this is due to local changes to the surrounding matrix (cooling rate in phantom gel and tumor microenvironment *in vivo*), which produce changes in the relative refractive index and presence of scatterers. Furtherrmore, we observed higher OAC along the length of the pathological *in vivo* images compared with normal, suggesting there are global changes in optical properties associated with pathological progression in the lungs.

### 3.7 Longitudinal Variation of Structure and Texture

**Fig. 17:**
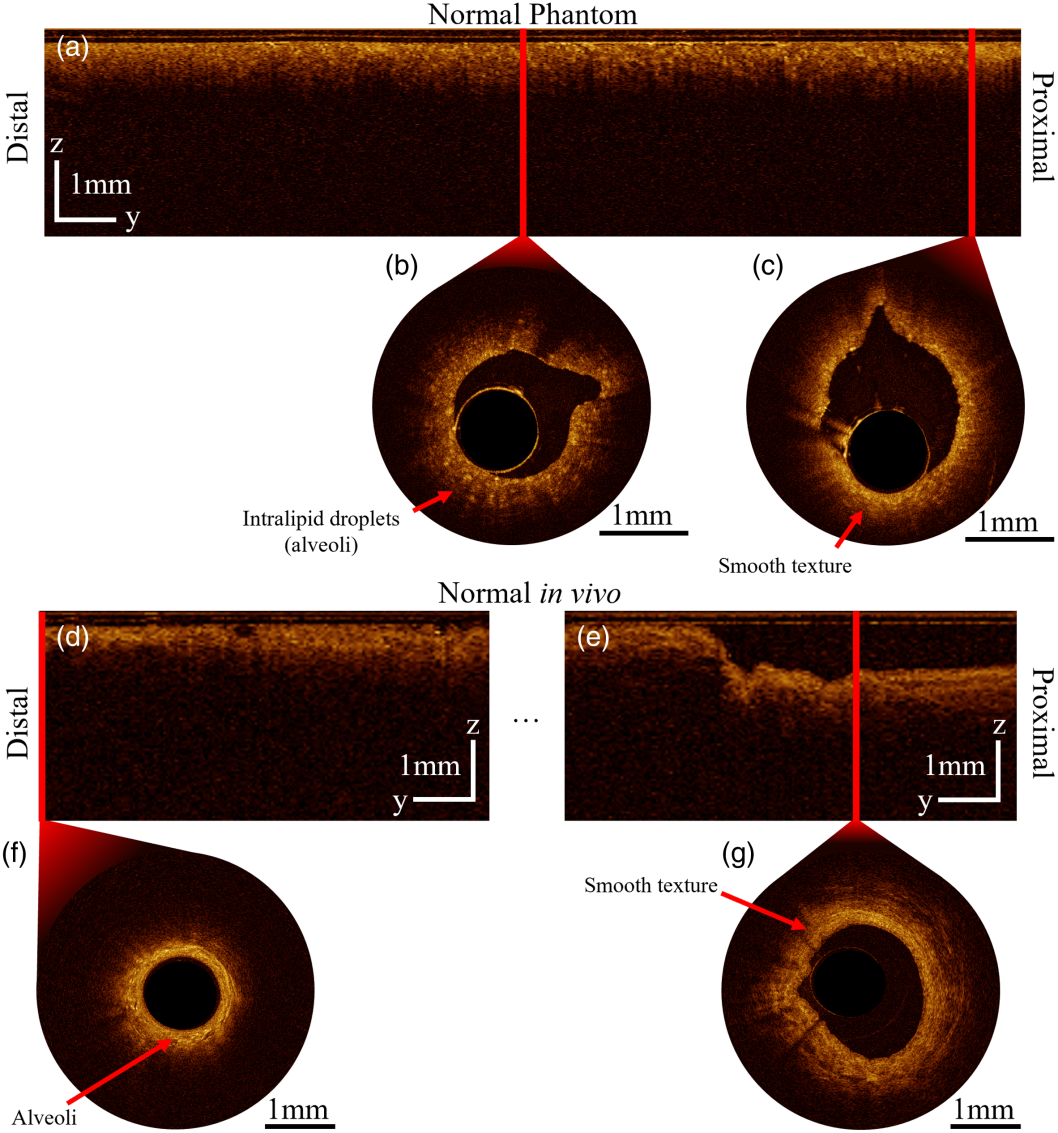
Comparison of B-scans from distal and proximal positions in the pullback direction for normal phantom (a, b, c) and normal segmental airway *in vivo* (d, e, f, g), with no smoothing applied (Video 1, MP4; [URL: https://doi.org/10.1117/1.JBO.30.10.105002.s1], 3.12 MB; Video 3, MP4, 3.95 MB [URL: https://doi.org/10.1117/1.JBO.30.10.105002.s3]). B-scans are displayed from distal and proximal portions of each longitudinal view (a, d, e), to visualize the presence of alveolar (b, f) structures and the transition to smooth texture proximally (c, g). Solid red lines indicate corresponding locations of the alternate view.

Longitudinal sections were generated from normal phantom and normal airway volumes to demonstrate the changes in texture and the density of alveoli observed in the pullback direction. Representative B-scans, from distal and proximal positions, are shown [Figs. 17(a), 17(d), and 17(e)], demonstrating textural changes at these positions. Smoother textures are visible in proximal airways (Figs. 17(c) and 17(g)] compared with distal [Figs. 17(b) and 17(f)], corresponding to transition of alveoli structures to smooth image texture. Furthermore, luminal diameter visibly increases relative to the probe surface, where a larger airgap is visible proximally than in more distal cross sections. Representative pullbacks of normal and lesion-containing cases in phantom and *in vivo* are provided in the Supplementary Material.

### 3.8 Phantom Angiography

**Fig. 18:**
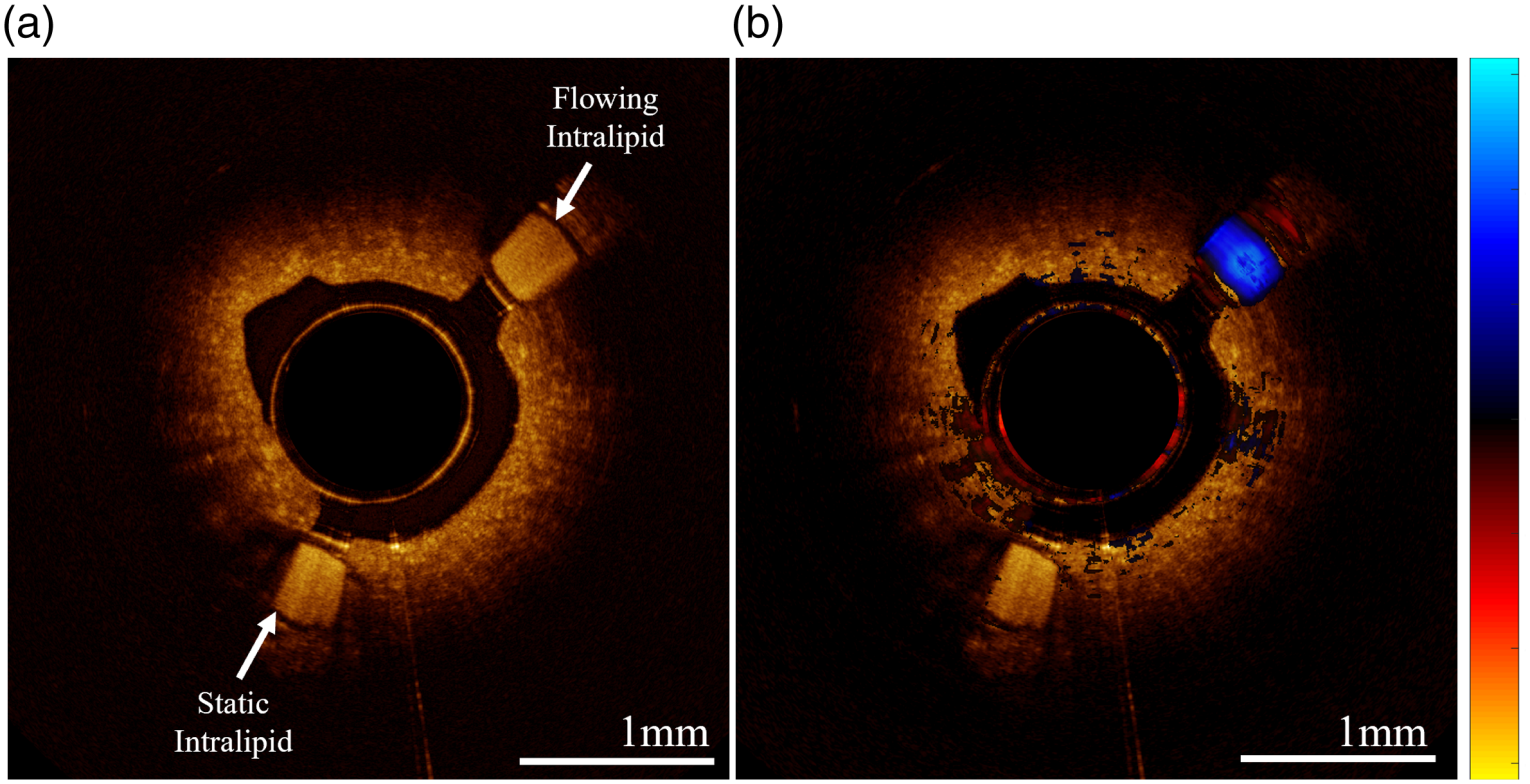
Cross-section of phantom with two embedded tubes containing static and 0.8 mL/min flowing 0.02g/mL intralipid. (a) OCT scan and (b) OCT overlayed inter-A-line Doppler angiogram (color bar units—π to π phase offset).

Doppler OCT demonstrates capability of differentiating the perfused and static intralipid from surrounding phantom matrix (Fig. 18). Tubing within the same embedded lesion phantom cross-section evaluated against *in vivo* is highlighted with an overlay indicating intralipid flow over structural OCT.

## 4 Discussion

This work demonstrates an endoscopic NIR OCT phantom that mimics optical behavior and morphology of human segmental airways. The phantom provides a platform for device characterization in absence of available biological specimens, particularly for the identification of embedded low-attenuation lesions. Through the use of OAC as a quantitative metric and qualitative image assessment, endoscopic OCT (1310±50  nm) of the presented agar phantom is demonstrated to be comparable to *in vivo* human lung tissue. Normal lung structures (e.g., alveoli, vessels, and smooth texture) as well as structural and optical markers of pathological progression (e.g., epithelial thickening) can be mimicked. In addition, the inclusion of tubing enables assessment of OCT angiography algorithms (e.g., Doppler OCT) in a tissue-mimicking environment.

### 4.1 Attenuation of Intralipid-Water Solutions

Transmission measurements at 1310 nm of uniform agar-intralipid-water gels show large increases in OAC when intralipid is cured with agar compared with intralipid-water solutions (Fig. 9). The effect is increased when the intralipid is added after boiling the agar-water solution compared with when added before, which we observed experimentally. This result differs from those collected at 650 nm in Ref. 32, which showed a decrease in intralipid’s OAC with agar gels, suggesting a wavelength dependence to the scattering response. Curiously, we observed that agar-water gels show near-zero OAC at 1310 nm; this suggests the observed OAC increase is due to an interaction of agar and intralipid. We hypothesize that differences in refractive index between the intralipid-agar gel and precipitate droplets (intralipid) produce highly scattering spherical objects that are not present in intralipid-water solutions. Previous studies have suggested these sharp changes can cause geometric errors in OCT due to refractive index changes at alveoli.^51^ The size of the intralipid spheres is determined by cooling rate of the gel, where faster cooling results in less intralipid precipitation and therefore smaller scattering objects. This behavior is reflected in the changes in image texture visible distal-to-proximal in the longitudinal sections (Fig. 17). Consequently, mold geometry and temperature control are critical for producing the desired imaging characteristics. Phantoms must be produced within 2 weeks of opening a new bag of intralipid, as we observed changes in precipitation behavior during storage once the seal has been punctured.

### 4.2 Repeatability

In accordance with the recommendations of Hacker et al.,^22^ our phantoms are assessed on eight characteristics: tissue-like properties, tunability, stability, architectural flexibility, reproducibility, simple maintenance, safety, and availability. Tissue-like properties are demonstrated through comparison of OAC and imaging characteristics to *in vivo* airway tissue. Tunability of OAC is achieved by adjusting the intralipid concentration (Figs. 10 and 14). As identified by Hacker, aqueous suspension phantoms (like the one presented in this work) have a limited shelf-life on the order of hours; however, we have performed a time series of measurements showing stability of the representative OAC for at least 6 h (Fig. 11). By using 3D printed molds, architectural flexibility is achieved, and additions or modifications to the phantom reagents can be readily implemented (Fig. 4). We demonstrated reproducibility to fabricate phantoms using a factorial experiment that investigated batch, sample, catheter, and pullback (factors) by measuring mode OAC (dependent variable) (Table 2). Overlap of the group-wise OAC histograms and 2.7% inter-batch variability in our repeatability experiment shows the capability of the method to produce phantoms with similar optical properties (Fig. 13). The phantoms can be maintained over their entire short shelf life in a 4°C refrigerator. All reagents used are biocompatible, nontoxic, and readily available. The agar-intralipid phantom presented in this work offers a different approach to tissue mimicry than the permanent silicone or rigid curing phantoms previously developed for OCT. The result is a simple and repeatable fabrication method at the expense of limited shelf life. Cost-effectiveness of phantoms produced using intralipid depends highly on integration of intralipid with an existing workflow within a research space, due to its high cost and relatively short shelf life; consequently, it may be cost prohibitive if the intralipid were used exclusively to produce phantoms.

### 4.3 Artifacts within OAC Measurements

A peaked intensity envelope is visible in all OCT A-lines, likely due to the focal position of the incident beam. These confocal effects are observed to be a source of error in this work, generating variations in measured intensity that is most apparent for samples with OAC >2 to $3\;{\mathrm{mm}}^{-1}$.^38^ Our findings are consistent with those of Cannon et al.^52^ who showed increased intensity from multiply scattering samples (such as intralipid), thus reducing the apparent OAC. In our previous work, we showed that higher order DCF inner-cladding modes result in the generation of multipath artifacts, which appear as axially displaced and dispersed “ghost” images in OCT.^53^ In this work, we set the reference arm of the OCT-AFI system such that the most prominent “ghost” is partially wrapped around the complex conjugate and does not interfere with our desired image [Figs. 16(c) and 16(d)]. However, less intense multipath artifacts appear with increased axial delays and can confound OAC measurements. Quantitatively, this appears as a reduction in the measured OAC within an image.

### 4.4 Comparison of Phantom versus *In Vivo* EB-OCT

The mode OAC of our normal phantom in the repeatability experiment differed by 16% from that of normal *in vivo* airway, demonstrating good simulation of the optical properties of segmental airway tissue (Fig. 15). The aim of the normal phantoms produced for the repeatability experiment was to show consistency between batches; however, a lower intralipid concentration could have been used to more closely match that of the presented *in vivo* cases. There are features present *in vivo* such as mucus, ducts, or emphysematous change that are not recreated in the presented phantom. Embedded simulated lesions in the phantom mimicked epithelial thickening resulting from an adenocarcinoma imaged *in vivo* (Fig. 16).

### 4.5 Phantom Angiography

The phantom angiography presented demonstrates how our tissue-mimicking phantom can also emulate realistic functional imaging. The successful discrimination between flowing and static intralipid serves as a proof-of-concept and further that flow within embedded tubing is visible through the phantom material (Fig. 18). Although intralipid is deployed for flow assessment, the small homogenous scatterers do not accurately represent the more forward scattering and relatively large biconcave disks of red blood cells. Anticoagulated animal blood could be used instead of intralipid, as desired, for future experiments. The mold could also be modified to allow embedded tubing in differing positions to assess depth penetration or directionality.

## 5 Conclusion

Using a solution of agar, intralipid, water, and coconut oil, the optical attenuation of *in vivo* human airway tissue is replicated. We mimic the appearance of morphologic features, such as alveoli and lumen diameter, along the length of the airway from distal to proximal, using a 3D printed mold with decreasing cross-sectional area and thermal mass. By embedding a simulated lesion in an otherwise normal gel phantom, we replicate attenuation contrast between adenocarcinoma and healthy tissue. Tubing is included in the phantom and demonstrated to be capable of representing blood flow within vasculature. The methodology is shown to be repeatable and match image characteristics of *in vivo* segmental airways as visualized by EB-OCT (including alveoli, luminal dilation, and epithelial thickening associated with pathologic progression), using qualitative (visualization of group-wise OAC histograms, comparison of OAC images) and quantitative (four-way ANOVA) analyses. The presented phantom demonstrates a lung-mimicking agar-intralipid gel that emulates the OAC contrast between normal and lesional tissue for device performance validation of endoscopic NIR OCT. This gel allows for improved device evaluation capabilities where biological specimens are unavailable and enables development of future phantoms with additional features or additives.

The phantom could be further developed through modeling larger regions of the lung, including luminal branching and proximal regions with cartilage rings. To achieve this, higher concentrations of agar would be needed to reduce physical damage during catheter insertion or exploration of other materials (such as Dragon Skin). There is also an opportunity to explore implementation of fluorescence contrast in the phantom, such that embedded lesions appear darker than surrounding tissue.

## Supplementary Material

10.1117/1.JBO.30.10.105002.s01

10.1117/1.JBO.30.10.105002.s1

10.1117/1.JBO.30.10.105002.s2

10.1117/1.JBO.30.10.105002.s3

10.1117/1.JBO.30.10.105002.s4

## Data Availability

Data and code developed in this study are available upon reasonable request to the corresponding author.

## References

[1] McLaughlin R. A. Noble P. B. Sampson D. D., “Optical coherence tomography in respiratory science and medicine: from airways to alveoli,” Physiology 29(5), 369–380 (2014). 10.1152/physiol.00002.2014 25180266

[r2] Hou R. et al., “Recent advances in optical coherence tomography for the diagnoses of lung disorders,” Expert Rev. Respir. Med. 5(5), 711–724 (2011). 10.1586/ers.11.59 21955240 PMC3393648

[r3] Kramer T. et al., “Advances in bronchoscopic optical coherence tomography and confocal laser endomicroscopy in pulmonary diseases,” Curr. Opin. Pulm. Med. 29(1), 11–20 (2023). 10.1097/MCP.0000000000000929 36474462 PMC9780043

[r4] Long H. et al., “EB-OCT: a potential strategy on early diagnosis and treatment for lung cancer,” Front. Oncol. 13, 1156218 (2023). FRTOA7 0071-9676 10.3389/fonc.2023.1156218 37182131 PMC10168178

[5] Pahlevaninezhad H. Lam S., “Optical coherence tomography: a review,” in Interventions in Pulmonary Medicine, Díaz-Jiménez J. P. Rodríguez A. N., Eds., pp. 379–391, Springer, Cham (2023).

[6] Breeze R. G. Wheeldon E. B., “The cells of the pulmonary airways,” Amer. Rev. Respir. Dis. 116(4), 705–777 (1977). ARDSBL 0003-0805 10.1164/arrd.1977.116.4.705 921054

[7] Knight D. A. Holgate S. T., “The airway epithelium: structural and functional properties in health and disease,” Respirology 8(4), 432–446 (2003). 10.1046/j.1440-1843.2003.00493.x 14708552

[8] Vaselli M. et al., “In vivo polarisation sensitive optical coherence tomography for fibrosis assessment in interstitial lung disease: a prospective, exploratory, observational study,” BMJ Open Respir. Res. 10(1), e001628 (2023). 10.1136/bmjresp-2023-001628 PMC10414088 37553184

[9] Ochs M. et al., “The number of alveoli in the human lung,” Amer. J. Respir. Crit. Care Med. 169(1), 120–124 (2004). AJCMED 1073-449X 10.1164/rccm.200308-1107OC 14512270

[10] Hariri L. P. et al., “Toward the guidance of transbronchial biopsy,” Chest 144(4), 1261–1268 (2013). CHETBF 0012-3692 10.1378/chest.13-0534 23828441 PMC3787917

[11] Lam S. et al., “In vivo optical coherence tomography imaging of preinvasive bronchial lesions,” Clin. Cancer Res. 14(7), 2006–2011 (2008). 10.1158/1078-0432.CCR-07-4418 18381938 PMC2849640

[r12] Hariri L. P. et al., “Diagnosing lung carcinomas with optical coherence tomography,” Ann. ATS 12(2), 193–201 (2015). 10.1513/AnnalsATS.201408-370OC PMC4342833 25562183

[r13] Hariri L. P. et al., “Volumetric optical frequency domain imaging of pulmonary pathology with precise correlation to histopathology,” Chest 143(1), 64–74 (2013). CHETBF 0012-3692 10.1378/chest.11-2797 22459781 PMC3537541

[r14] Michel R. G. et al., “Optical coherence tomography as an adjunct to flexible bronchoscopy in the diagnosis of lung cancer: a pilot study,” Chest 138(4), 984–988 (2010). CHETBF 0012-3692 10.1378/chest.10-0753 20472863

[r15] Tsuboi M. et al., “Optical coherence tomography in the diagnosis of bronchial lesions,” Lung Cancer 49(3), 387–394 (2005). 10.1016/j.lungcan.2005.04.007 15922488

[16] Zhu Q. et al., “Novel image features of optical coherence tomography for pathological classification of lung cancer: results from a prospective clinical trial,” Front. Oncol. 12, 870556 (2022). FRTOA7 0071-9676 10.3389/fonc.2022.870556 36338729 PMC9634220

[17] Cannon T. M. Bouma B. E. Uribe-Patarroyo N., “Layer-based, depth-resolved computation of attenuation coefficients and backscattering fractions in tissue using optical coherence tomography,” Biomed. Opt. Express 12(8), 5037–5056 (2021). BOEICL 2156-7085 10.1364/BOE.427833 34513241 PMC8407832

[r18] Liu J. et al., “Optimized depth-resolved estimation to measure optical attenuation coefficients from optical coherence tomography and its application in cerebral damage determination,” J. Biomed. Opt. 24(3), 035002 (2019). JBOPFO 1083-3668 10.1117/1.JBO.24.3.035002 30834722 PMC6975193

[r19] Chang S. Bowden A. K., “Review of methods and applications of attenuation coefficient measurements with optical coherence tomography,” J. Biomed. Opt. 24(9), 090901 (2019). JBOPFO 1083-3668 10.1117/1.JBO.24.9.090901 31520468 PMC6997582

[20] Vermeer K. A. et al., “Depth-resolved model-based reconstruction of attenuation coefficients in optical coherence tomography,” Biomed. Opt. Express 5(1), 322–337 (2013). BOEICL 2156-7085 10.1364/BOE.5.000322 24466497 PMC3891343

[21] Pogue B. W. Patterson M. S., “Review of tissue simulating phantoms for optical spectroscopy, imaging and dosimetry,” J. Biomed. Opt. 11(4), 041102 (2006). JBOPFO 1083-3668 10.1117/1.2335429 16965130

[22] Hacker L. et al., “Criteria for the design of tissue-mimicking phantoms for the standardization of biophotonic instrumentation,” Nat. Biomed. Eng. 6(5), 541–558 (2022). 10.1038/s41551-022-00890-6 35624150

[23] Pifferi A. et al., “Performance assessment of photon migration instruments: the MEDPHOT protocol,” Appl. Opt. 44(11), 2104–2114 (2005). APOPAI 0003-6935 10.1364/AO.44.002104 15838951

[24] Lamouche G. et al., “Review of tissue simulating phantoms with controllable optical, mechanical and structural properties for use in optical coherence tomography,” Biomed. Opt. Express 3(6), 1381–1398 (2012). BOEICL 2156-7085 10.1364/BOE.3.001381 22741083 PMC3370977

[25] Listewnik P. et al., “Porous phantoms mimicking tissues—investigation of optical parameters stability over time,” Materials 14(2), 423 (2021). MATEG9 1996-1944 10.3390/ma14020423 33467152 PMC7829841

[26] Goldfain A. M. et al., “Polydimethylsiloxane tissue-mimicking phantoms with tunable optical properties,” J. Biomed. Opt. 27(7), 074706 (2022). JBOPFO 1083-3668 10.1117/1.JBO.27.7.074706 PMC8601433 34796707

[27] Baxi J. et al., “Retina-simulating phantom for optical coherence tomography,” J. Biomed. Opt. 19(2), 021106 (2013). JBOPFO 1083-3668 10.1117/1.JBO.19.2.021106 24042445

[28] Jenne S. Zappe H., “Multiwavelength tissue-mimicking phantoms with tunable vessel pulsation,” J. Biomed. Opt. 28(4), 045003 (2023). JBOPFO 1083-3668 10.1117/1.JBO.28.4.045003 37077500 PMC10109273

[29] Ntombela L. Adeleye B. Chetty N., “Low-cost fabrication of optical tissue phantoms for use in biomedical imaging,” Heliyon 6(3), e03602 (2020). 10.1016/j.heliyon.2020.e03602 32258463 PMC7096755

[30] Pilvar A. et al., “Shortwave infrared spatial frequency domain imaging for non-invasive measurement of tissue and blood optical properties,” J. Biomed. Opt. 27(6), 066003 (2022). JBOPFO 1083-3668 10.1117/1.JBO.27.6.066003 35715883 PMC9204261

[31] van Staveren H. J. et al., “Light scattering in lntralipid-10% in the wavelength range of 400–1100 nm,” Appl. Opt. 30(31), 4507–4514 (1991). APOPAI 0003-6935 10.1364/AO.30.004507 20717241

[32] Cubeddu R. et al., “A solid tissue phantom for photon migration studies,” Phys. Med. Biol. 42(10), 1971 (1997). PHMBA7 0031-9155 10.1088/0031-9155/42/10/011 9364593

[33] Kim M. et al., “Fabrication of agar-based tissue-mimicking phantom for the technical evaluation of biomedical optical imaging systems,” Curr. Appl. Phys. 61, 80–85 (2024).1567-1739 10.1016/j.cap.2024.02.013

[34] Zulina N. et al., “Colon phantoms with cancer lesions for endoscopic characterization with optical coherence tomography,” Biomed. Opt. Express 12(2), 955–968 (2021). BOEICL 2156-7085 10.1364/BOE.402081 33680552 PMC7901311

[35] Smith G. T. et al., “Multimodal 3D cancer-mimicking optical phantom,” Biomed. Opt. Express 7(2), 648–662 (2016). BOEICL 2156-7085 10.1364/BOE.7.000648 26977369 PMC4771478

[36] Durkee M. S. et al., “Fabrication and characterization of optical tissue phantoms containing macrostructure,” J. Vis. Exp. 12(132), 57031 (2018). 10.3791/57031 PMC5912403 29553502

[37] Flock S. T. et al., “Optical properties of intralipid: a phantom medium for light propagation studies,” Lasers Surg. Med. 12(5), 510–519 (1992). LSMEDI 0196-8092 10.1002/lsm.1900120510 1406004

[38] Raizada R., Identification of Qualitative and Quantitative Features in Wide-field in vivo Oral Optical Coherence Tomography, University of British Columbia (2018).

[39] Liu H.-C. et al., “Intraoperative application of optical coherence tomography for lung tumor,” J. Biophotonics 16(6), e202200344 (2023). 10.1002/jbio.202200344 36755475

[40] Ding M. et al., “Optical coherence tomography for identification of malignant pulmonary nodules based on random forest machine learning algorithm,” PLoS One 16(12), e0260600 (2021). POLNCL 1932-6203 10.1371/journal.pone.0260600 34971557 PMC8719667

[41] Lee A. M. D. et al., “Fiber-optic polarization diversity detection for rotary probe optical coherence tomography,” Opt. Lett. 39(12), 3638–3641 (2014). OPLEDP 0146-9592 10.1364/OL.39.003638 24978556

[42] Pahlevaninezhad H. et al., “A high-efficiency fiber-based imaging system for co-registered autofluorescence and optical coherence tomography,” Biomed. Opt. Express 5(9), 2978–2987 (2014). BOEICL 2156-7085 10.1364/BOE.5.002978 25401011 PMC4230860

[43] Fisher R. A., “Statistical methods for research workers,” in Breakthroughs in Statistics, Kotz S. Johnson N. L., Eds., pp. 66–70, Springer, New York, NY (1992).

[44] Peters C. M. et al., “Airway luminal area and the resistive work of breathing during exercise in healthy young females and males,” J. Appl. Physiol. 131(6), 1750–1761 (2021). 10.1152/japplphysiol.00418.2021 34709072

[45] Pahlevaninezhad H. et al., “Endoscopic Doppler optical coherence tomography and autofluorescence imaging of peripheral pulmonary nodules and vasculature,” Biomed. Opt. Express 6(10), 4191 (2015). BOEICL 2156-7085 10.1364/BOE.6.004191 26504665 PMC4605074

[46] Whitmore R., Rheology of the Circulation, 1st ed., Pergamon Press, Oxford (1968).

[47] Lee A. M. D. et al., “In vivo lung microvasculature visualized in three dimensions using fiber-optic color Doppler optical coherence tomography,” J. Biomed. Opt. 18(5), 050501 (2013). JBOPFO 1083-3668 10.1117/1.JBO.18.5.050501 23625308

[48] Dreischuh T. et al., “Turbid media extinction coefficient for near-infrared laser radiation,” J. Phys.: Conf. Ser. 594(1), 012030 (2015). JPCSDZ 1742-6588 10.1088/1742-6596/594/1/012030

[49] Palmer K. F. Williams D., “Optical properties of water in the near infrared*,” J. Opt. Soc. Amer. 64(8), 1107–1110 (1974). 10.1364/JOSA.64.001107

[50] Wieliczka D. M. Weng S. Querry M. R., “Wedge shaped cell for highly absorbent liquids: infrared optical constants of water,” Appl. Opt. 28(9), 1714–1719 (1989). APOPAI 0003-6935 10.1364/AO.28.001714 20548731

[51] Golabchi A. et al., “Refractive errors and corrections for OCT images in an inflated lung phantom,” Biomed. Opt. Express 3(5), 1101–1109 (2012). BOEICL 2156-7085 10.1364/BOE.3.001101 22567599 PMC3342185

[52] Cannon T. M. Bouma B. E. Uribe-Patarroyo N., “Mapping optical scattering properties to physical particle information in singly and multiply scattering samples,” Biomed. Opt. Express 14(8), 4326–4348 (2023). BOEICL 2156-7085 10.1364/BOE.494518 37799686 PMC10549752

[53] Tanskanen A. et al., “Higher-order core-like modes in double-clad fiber contribute to multipath artifacts in optical coherence tomography,” J. Lightwave Technol. 39(17), 5573–5581 (2021). JLTEDG 0733-8724 10.1109/JLT.2021.3088055

[54] Fung A. et al., “Segmental airway phantom for endobronchial optical coherence tomography,” Proc. SPIE 12833, 1283306 (2024). PSISDG 0277-786X 10.1117/12.3003437

