# Supplementary material for: Segmental airway adenocarcinoma-simulating phantom for endoscopic near-infrared optical coherence tomography
Source: J Biomed Opt. 2025 Oct 7;30(10):105002. doi: 10.1117/1.JBO.30.10.105002 (PMC12509969; doi:10.1117/1.JBO.30.10.105002)
Supplement: Supplementary file 1 [file JBO_030_105002_SD001.pdf]

Supplemental Material
Segmental airway phantom preparation procedure

Equipment

- Hot plate
- Thermometer
- 2x250 mL borosilicate beaker
- 50 mL graduated cylinder
- Top loading balance
- Disposable scoop
- 18 G 1” blunt needle
- 26 G 1/2” blunt needle
- 3x5mL syringe
- 2x5cm 0.021”ID, 0.003” Wall PET tubing (021-0030 PET01. Nordson Medical. Salem, New Hampshire)
- Swab
- Tweezers
- Scalpel
- 1/8” round precision file
- UV-cure epoxy (NOA 63. Norland Products Inc. Jamesburg, New Jersey)
- UV curing lamp (Liquid light guide allows for improved flexibility)
- Refrigerator (4°C)

Reagents

- Agar powder (05039 Agar. MilliporeSigma Canada Ltd. Oakville, Canada)
- Intralipid (Intralipid® 20%. Fresenius Kabi Canada Ltd. Toronto, Canada)
- Coconut oil (Organic virgin coconut oil, cold pressed & unrefined. Nutiva. Richmond, California)
- Deionized water

Table 1. Materials for normal and lesion lung tissue mimicking phantom solutions using OCT at 1310 nm

Reagent	Normal	Lesion
Agar powder	0.5 g	0.5 g
Coconut Oil	3.0 mL	3.0 mL
Intralipid®20%	2 mL	0.5 mL
Deionized Water	45 mL	45 mL
Total	50.5 mL	49 mL

Procedure

Prepare 4 moulds.

1. 3D print moulds using filament printer
    a. STL available at: https://github.com/cancer-imaging/Lung-Phantom-Mold
2. Examine lumen cone to ensure matrix will not be damaged when cone is extracted.
3. File internal surfaces to remove “stringing”.
4. Using dental pick, open holes in bottom of mould to ensure PET tubing can be inserted.
5. Rasp front edges of tubing supports to remove burrs.
6. With UV cure epoxy:
    a. Insert PET tubing into hole, position against support, with ~ 1cm extending out either end of mould
    b. Tack tube in position with small amount of epoxy and cure epoxy.
    c. Secure in position, applying epoxy along length of tube and cure epoxy.
    d. Seal around perimeter of tube on external bottom face of mould (so matrix cannot escape via tube port) and cure epoxy.

Prepare matrix:

7. Heat up hotplate to 300ºC.
8. Using scale and disposable scoop, tare to beaker #1 and dispense 0.5 g of agar.
9. Scoop ~5 mL of coconut oil into beaker #2, place on hotplate, stir until melted. Fill syringe #1 with 3 mL coconut oil.
10. Dispense 45 mL of deionized water into graduated cylinder.
11. Attach 18 G 1" blunt needle tip to syringe #2; fill with intralipid from IV bag (refer to table 1; if implanting lesion use 0.5 mL or for normal matrix use 2 mL).
12. Combine coconut oil, distilled water, intralipid into beaker #1 containing the agar.
13. Heat beaker #1 to 95ºC, stirring intermittently, then remove from hot plate.
14. If implanting lesion:
    a. Pull ~2 mL of lesion solution into syringe #3.
    b. Place small droplets on lumen cone.
    c. Return remaining solution in syringe to beaker #1.
    d. Place moulds into fridge for 5 minutes.
    e. Add remaining intralipid (1.5 mL) to beaker to create normal matrix solution.
    f. Reheat to 80ºC then remove from heat.
15. When mixture cooled to 60ºC, extract into syringe #3.
16. Dispense matrix into moulds (should fill 4 moulds). If needle tip becomes blocked, immerse in beaker 1 to remelt.
17. Place in fridge immediately.
18. Allow to cure for >4 hours or preferably overnight before imaging. Phantoms maintain their properties for approximately 48 hours from when they are first placed in the fridge.

Prepare phantoms for imaging:

19. Use scalpel to cut around perimeter of lumen at bottom face, being careful to not damage tubing.
20. Use tweezers to push lumen cone out top surface of matrix, then extract, being careful not to damage matrix.
21. Insert 26 G ½" blunt needle into one tube, epoxy into position using Norland 63.

Table 2. Record of phantom batches

Batch #	Description	Sample Number			
Batch start time:		Placed in fridge:			
Total Samples					

Table 3. Record of Images collected

Batch:	Time removed from fridge:				
Sample	Probe #	Imaging Time	Pullback #		
	Time returned to fridge:				
